# Wound Healing Potential of Multifunctional Nanomaterials: Mechanism, Future Prospects, and Challenges

**DOI:** 10.3390/pharmaceutics18091054

**Published:** 2026-08-25

**Authors:** Akshay Kumar, Devesh Kumar, Mohit Agrawal, Jaspreet Kaur, Mohit Kumar, Dinesh Kumar, Neeraj Choudhary, Thakur Gurjeet Singh, Ankit Awasthi, Emad M. Abdallah

**Affiliations:** 1 Chitkara College of Pharmacy, Chitkara University, Rajpura 140401, Punjab, India; 2 School of Medical & Allied Sciences, K.R. Mangalam University, Gurugram 122103, Haryana, India; 3 Department of Food Processing Technology, Sri Guru Granth Sahib World University (SGGSWU), Fatehgarh Sahib 140407, Punjab, India; 4 GNA School of Pharmacy, GNA University, Phagwara 144401, Punjab, India; dineshpotlia123@gmail.com (D.K.);; 5 Department of Biology, College of Science, Qassim University, Buraydah 51452, Saudi Arabia

**Keywords:** multifunctional nanomaterials, scar-free wound healing, antimicrobial activity, nanoparticles, tissue regeneration, signaling pathways, regenerative nanomedicine

## Abstract

Wound healing is a dynamic and highly coordinated process that involves inflammation, cell proliferation, angiogenesis, re-epithelialization, extracellular matrix remodeling, and tissue maturation. The altered expression of important signaling pathways, such as transforming growth factor-β (TGF-β)/Smad, nuclear factor-κB (NF-κB), phosphoinositide 3-kinase/protein kinase B (PI3K/Akt), mitogen-activated protein kinase (MAPK), and Wnt/β-catenin, may be responsible for slower wound healing, chronic inflammation, excessive fibrosis, and impaired tissue regeneration. Multifunctional nanomaterials are a promising strategy for tuning these highly coordinated processes due to their tunable physicochemical properties, high surface area, and the ability to deliver cargo, as well as the integration of antimicrobial, antioxidant, anti-inflammatory, and pro-angiogenic properties. The aim of current review is to summarize the potential of multifunctional nanomaterials to promote wound healing, with a focus on mechanisms of action and modulation of key cellular signaling pathways. A systematic review of the literature was conducted using PubMed, Scopus, Web of Science, and Google Scholar, searching for publications from 1996 to June 2026, and representative experimental, mechanistic, preclinical, and translational studies were critically evaluated. In this review, the authors discuss the role of nanomaterial properties, therapeutic payload, molecular targets, modulation of cellular signaling pathways, and regenerative effects. These platforms have been shown in in vitro and animal studies to influence inflammatory signaling, oxidative stress, angiogenesis, collagen remodeling, re-epithelialization, cellular proliferation, and migration. However, the modulation of these pathways are dose-responsive, time-dependent, and cell- and wound-stage-specific. Despite the promising therapeutic potential of nanomaterial-based wound care strategies, the available evidence remains predominantly preclinical, with relatively limited clinical data supporting their use in humans. Concerns regarding long-term toxicity, biodistribution, batch-to-batch reproducibility, sterilization, scalable manufacturing, regulatory approval, and commercial feasibility further challenge translation into clinical practice. Multifunctional nanomaterials may offer a promising approach for pathway-specific and multimodal wound management; however, comprehensive mechanistic studies, long-term safety and biodistribution assessments, and well-designed clinically relevant investigations are required to establish their efficacy, safety, and true translational potential.

## 1. Introduction

Skin injuries and pathological scarring represent a major global health burden, with millions of patients annually suffering from delayed or impaired healing, such as diabetic foot ulcers, pressure ulcers, and venous leg ulcers [1,2]. In addition to pathological scarring that occurs in 1–2% of the global population, scarring can be accompanied by excessive collagen deposition, fibroblast hyperproliferation, and persistent inflammation (known as hypertrophic scars and keloids). Acute wounds resulting from trauma, burns, and surgical procedures can also fail to heal properly, and pathological scarring can occur following healing [3,4]. These conditions not only impair the integrity and mechanical function of the skin but also result in cosmetic disfigurement, pain, and psychological distress. Wound healing is a highly dynamic and tightly regulated biological process that can be divided into four overlapping phases: haemostasis, inflammation, proliferation, and remodeling [5]. Haemostasis is characterized by platelet activation and fibrin clot formation to prevent blood loss and provide a provisional matrix for cell migration. The inflammatory phase is characterized by recruitment of neutrophils and macrophages to clear debris and pathogens and by the release of cytokines and growth factors. After the inflammatory phase, the proliferative phase stimulates fibroblast proliferation and migration, ECM deposition, angiogenesis to form granulation tissue, and re-epithelialization [6]. In the final phase, remodeling, collagen III is replaced by collagen I and structural reorganisation of the ECM restores tissue strength, but dysregulated scar formation can occur due to sustained inflammation and aberrant fibroblast activity that results in excessive ECM deposition. Some of the key molecular signaling pathways involved in wound healing and scar formation are the transforming growth factor-β (TGF-β)/Smad pathway, which mediates fibroblast activation and collagen synthesis; the Wnt/β-catenin pathway, which regulates cell proliferation and tissue regeneration; the PI3K/Akt pathway, which controls cell survival, migration, and angiogenesis; and the MAPK/ERK pathway, which controls cell proliferation and migration [7]. Moreover, pathways like NF-κB, HIF-1α/VEGF, and Notch signaling are important for inflammation, oxidative stress responses, and neovascularization in wound repair. Although much progress has been made in wound management, traditional therapies, such as gauze dressings, topical antimicrobials, and polymeric wound coverings, offer passive protection and are unable to actively regulate the intricate biochemical and cellular environment of wounds due to their limitations, such as poor drug penetration, uncontrolled drug release, inadequate antibacterial activity, and limited ability to modulate inflammatory and regenerative signaling pathways. Multifunctional nanomaterials possessing several physicochemical properties including nanoscale size, high surface-to-volume ratio, tunable surface chemistry [8], and improved interaction with biological systems have been explored as a promising strategy in regenerative medicine, and various nanomaterials, such as metallic nanoparticles (e.g., silver, gold, and zinc oxide), polymeric nanoparticles, nanofibers, nanogels, and nanocomposite hydrogels, have shown great potential in promoting wound healing by providing antimicrobial activity [9,10], reactive oxygen species (ROS) scavenging, immunomodulation, and controlled delivery of therapeutic agents (e.g., growth factors, nucleic acids, and anti-inflammatory drugs) that can actively modulate key cellular signaling pathways involved in tissue repair (e.g., TGF-β/Smad-mediated collagen remodeling, PI3K/Akt-driven angiogenesis, NF-κB-regulated inflammatory responses, and HIF-1α-dependent vasculogenesis) [6].

In addition, novel nanomaterial-based platforms, including stimuli-responsive systems, bioinspired nanocomposites, and smart microneedle-assisted delivery systems, are being developed to improve drug penetration, achieve spatial and temporal control of therapeutic release, and imitate the natural extracellular matrix. This review summarises recent progress in multifunctional nanomaterials for improving wound healing, with an emphasis on material design strategies, biological mechanisms, and modulation of critical signaling pathways involved in wound healing, as well as current challenges, translational opportunities, and future research perspectives for the clinical application of nanomaterial-based regenerative therapies.

### Literature Search Strategy

A comprehensive literature search was conducted to identify relevant studies on nanomaterial-based approaches for skin wound healing, tissue regeneration, and the associated molecular signaling mechanisms. The search was performed using PubMed, Scopus, Web of Science, and Google Scholar databases. The search strategy was developed using combinations of keywords related to nanomaterials, wound healing, and signaling pathways. The principal Boolean search string was: (“nanomaterial” OR “nanoparticle” OR “metal nanoparticle” OR “metal oxide nanoparticle” OR “polymeric nanoparticle” OR “chitosan nanoparticle” OR “carbon-based nanomaterial” OR “nanofiber” OR “nanogel” OR “hydrogel” OR “lipid-based nanocarrier” OR “nanocomposite”) AND (“skin wound healing” OR “wound healing” OR “chronic wound” OR “diabetic wound” OR “burn wound” OR “scar-free healing” OR “tissue regeneration”) AND (“signalling pathway” OR “signalling pathway” OR “TGF-β/Smad” OR “PI3K/Akt” OR “NF-κB” OR “MAPK” OR “Wnt/β-catenin” OR “JAK/STAT” OR “Nrf2” OR “HIF-1α/VEGF” OR “angiogenesis” OR “inflammation” OR “fibrosis”). Additional combinations were used where required to identify studies addressing specific nanomaterial classes or individual signaling pathways. The literature search covered publications from 1996 to June 2026, with the final search conducted in June 2026, to capture foundational studies describing wound healing and scar formation and recent advances in multifunctional nanomaterials and pathway-directed therapeutic strategies. Studies were considered for inclusion when they provided relevant experimental, mechanistic, preclinical, or translational evidence concerning nanomaterials for skin wound healing or scar reduction, particularly when they investigated inflammation, oxidative stress, antimicrobial activity, angiogenesis, cell proliferation and migration, re-epithelialization, extracellular matrix remodeling, collagen deposition, fibrosis, or modulation of specific signaling pathways. Original research articles were prioritized, while relevant review articles and landmark studies were included to establish fundamental concepts and provide mechanistic context. Studies unrelated to cutaneous wound healing, duplicate publications, studies focused exclusively on non-wound applications, and publications lacking sufficient information regarding the nanomaterial, therapeutic mechanism, or biological outcome were excluded. As the present work is a narrative review rather than a systematic review or meta-analysis, no formal quantitative synthesis was performed. Instead, representative studies were selected according to their relevance to the review scope, mechanistic significance, quality of experimental evidence, novelty of the nanomaterial platform, and translational importance. Particular emphasis was placed on studies that established a mechanistic relationship between nanomaterial characteristics or therapeutic cargo and key pathways involved in inflammation, angiogenesis, proliferation, tissue remodeling, and fibrosis. The selected literature was subsequently organized according to the major nanomaterial classes and their corresponding wound-healing mechanisms and signaling pathways.

## 2. Mechanisms of Scar Formation in Skin Wound Healing

**Figure 1:**
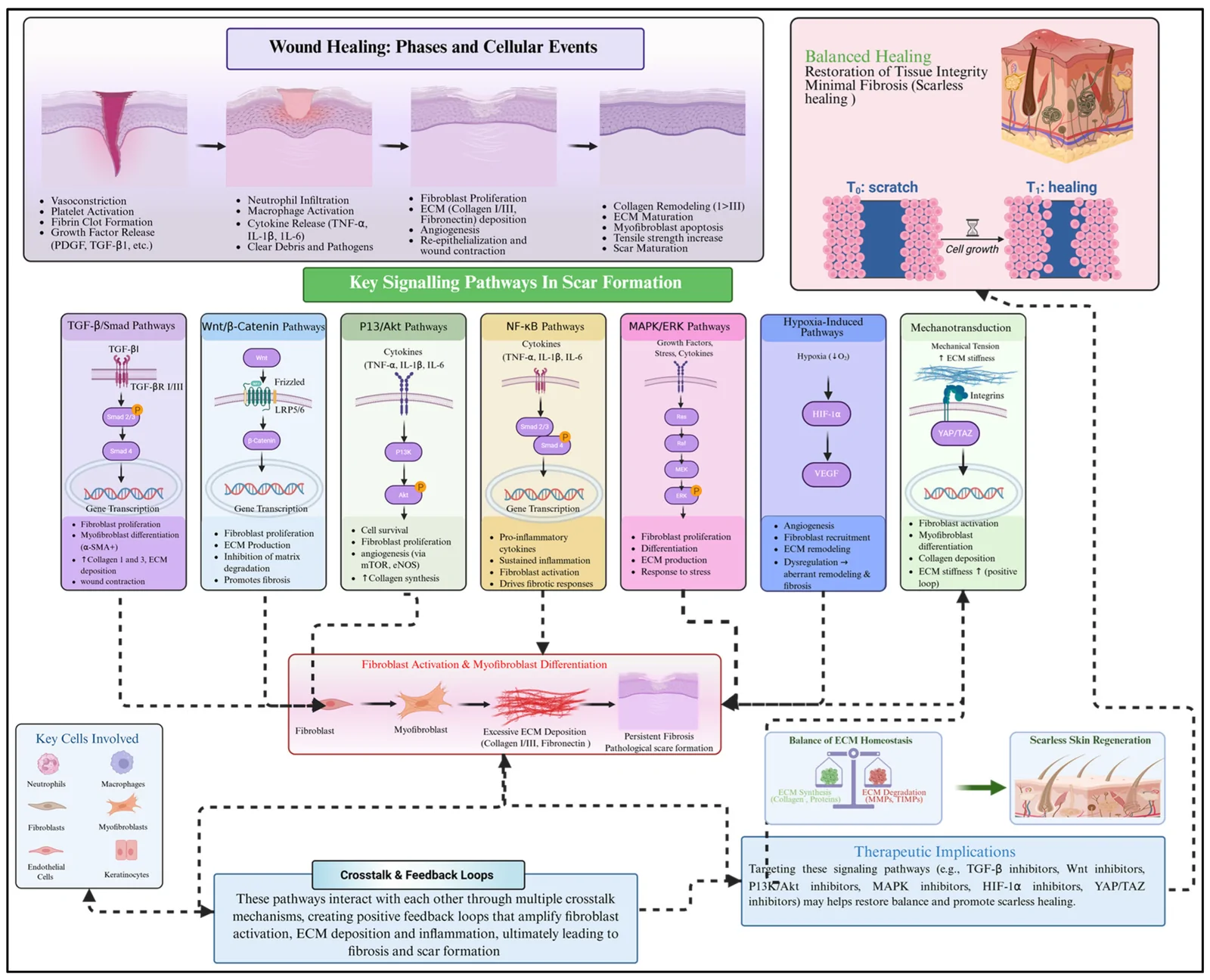
Molecular signaling networks linking wound healing to pathological scar formation. Overview of the major cellular events and interconnected signaling pathways regulating wound repair, fibroblast activation, extracellular matrix remodeling, and progression toward either balanced healing or pathological fibrosis.

Skin wound healing is a complex biological process that occurs in a regulated, sequential, and well-coordinated manner under physiological conditions, involving a tightly regulated balance between hemostasis, inflammation, proliferation, and remodeling that allows the restoration of tissue integrity and minimal fibrosis [11,12,13]. Dysregulation of the tissue repair cascade can cause excessive ECM deposition and abnormal tissue remodeling, leading to the formation of pathological scars, such as hypertrophic scars and keloids [14,15]. The transforming growth factor-β (TGF-β)/Smad signaling pathway, particularly TGF-β1, is one of the major molecular regulators of scar formation through stimulating fibroblast proliferation and differentiation into myofibroblasts, which produce excess collagen types I and III; myofibroblasts are also the major players in wound contraction and ECM deposition, but persistent activation of the pathway results in fibrotic tissue accumulation and thickened scar formation [16,17]. The Wnt/β-catenin signaling pathway also controls fibroblast proliferation and matrix remodeling; abnormal activation of this pathway has been linked to elevated fibrosis and excessive scar tissue formation [18,19]. The PI3K/Akt signaling pathway also modulates cell survival, proliferation, and angiogenesis during wound healing, and its prolonged activation may increase fibroblast activity and collagen production [20,21]. In addition, inflammatory signaling pathways like NF-κB that regulate pro-inflammatory cytokines like TNF-α, IL-1β, and IL-6 can sustain the inflammatory phase and drive fibrotic responses [22], while the mitogen-activated protein kinase (MAPK/ERK) pathway that controls cellular proliferation, differentiation, and stress responses in dermal fibroblasts is another important signaling pathway that is active in the wound microenvironment [23]. In addition, hypoxia in the wound microenvironment is a critical factor that activates the hypoxia-inducible factor-1α (HIF-1α)/vascular endothelial growth factor (VEGF) pathway [24], which promotes angiogenesis and fibroblast recruitment but can also lead to aberrant tissue remodeling if dysregulated. In addition, mechanical tension and changes in ECM stiffness can trigger mechanotransduction pathways, such as YAP/TAZ signaling [25,26], which stimulate fibroblast activation and collagen deposition, adding to the imbalance in ECM synthesis and degradation that perpetuates persistent fibrosis and scar formation. These signaling pathways and cellular interactions will be important for the future development of more advanced therapeutic approaches to modulate wound-healing processes and promote scarless skin regeneration. Figure 1 shows the signaling pathways of scar wound healing.

## 3. Design Principles of Multifunctional Nanomaterials for Improved Wound Healing

**Figure 2:**
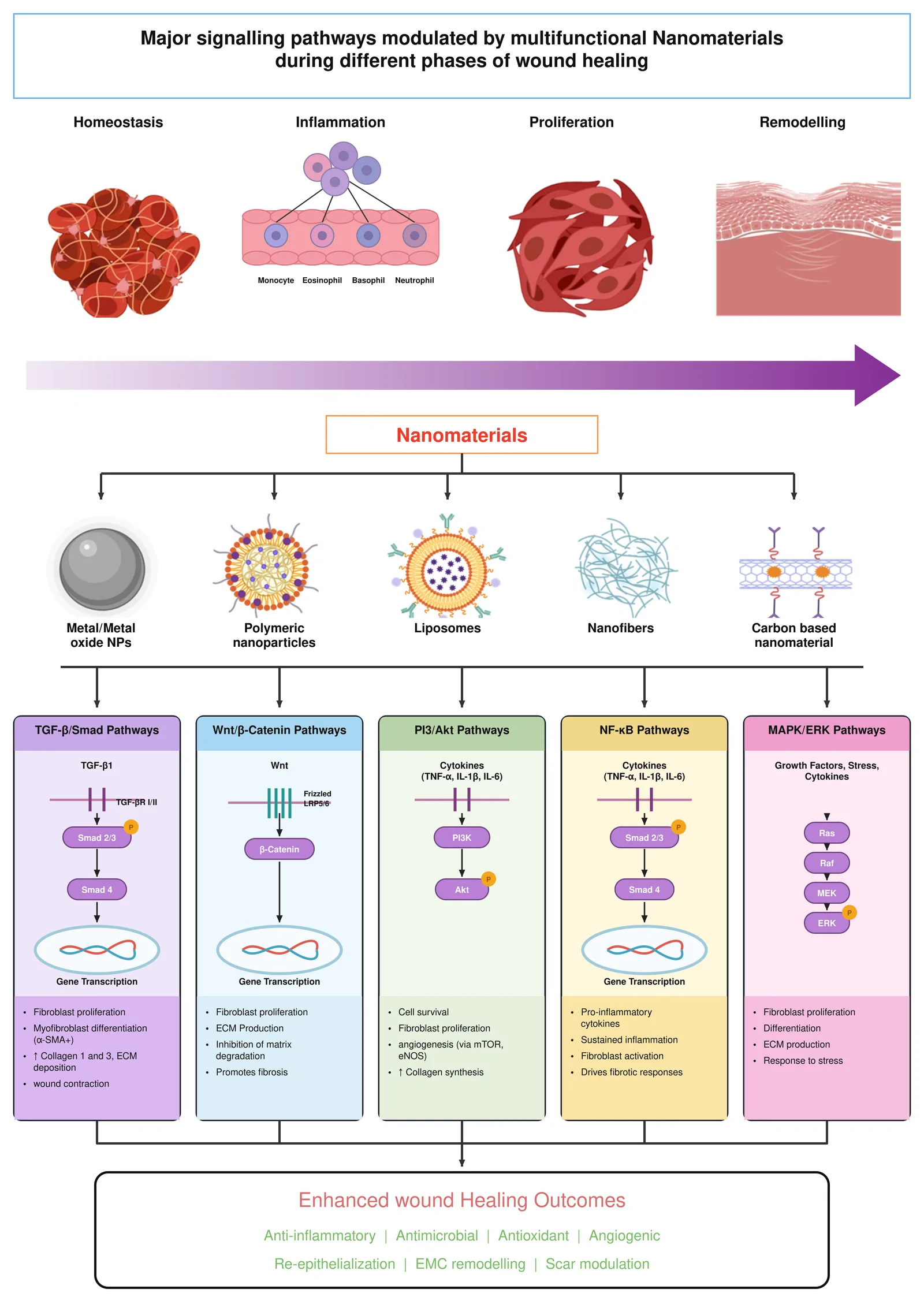
Multifunctional nanomaterials and signaling pathways across the phases of wound healing. Representative nanomaterial platforms can influence TGF-β/Smad, Wnt/β-catenin, PI3K/Akt, NF-κB, and MAPK signaling, thereby affecting inflammation, cell proliferation, angiogenesis, re-epithelialization, and extracellular matrix remodeling.

Multifunctional nanomaterials for improved wound healing and skin regeneration must be designed to address the complex biological environment of wounds and the multiple factors that regulate tissue repair [27]. These include key pathological processes in impaired healing and scar formation, such as infection, excessive inflammation, oxidative stress, and abnormal ECM remodeling [28,29]. The first design principle involves regulating the physicochemical properties of nanomaterials, such as particle size, surface charge, morphology, and surface functionalization, to control cellular uptake, tissue penetration, and interactions with biological components [30,31]. Nanomaterials with nanoscale dimensions offer a high surface-to-volume ratio, which facilitates the efficient loading and controlled delivery of therapeutic agents (e.g., growth factors, anti-inflammatory drugs, nucleic acids, and antioxidants), and surface modification with biocompatible polymers, peptides, or bioactive ligands improves stability, targeted delivery, and cellular interactions with the wound microenvironment [32]. In contrast, the incorporation of multifunctionality to provide antibacterial activity, reactive oxygen species (ROS) scavenging, immunomodulation, and angiogenesis promotion is another important design strategy [33]. For example, metallic nanoparticles like silver, gold, and zinc oxide can exhibit powerful antimicrobial properties [34]. In contrast, polymeric nanocarriers and nanocomposite hydrogels can be used to deliver bioactive molecules in a controlled and sustained manner. Stimuli-responsive nanomaterials respond to environmental cues such as pH, temperature, enzymes, or reactive oxygen species for on-demand drug release [35] and adaptive therapeutic responses to dynamic wound environments, and biomimetic design approaches replicate the structure and function of the natural extracellular matrix for improved cell adhesion, proliferation, and tissue regeneration [36,37]. Notably, multifunctional nanomaterials can be engineered to modulate key molecular signaling pathways involved in wound healing and scar formation, such as the TGF-β/Smad pathway for collagen synthesis, the PI3K/Akt pathway for cell survival and angiogenesis, and the NF-κB pathway for inflammatory responses, to promote balanced tissue remodeling and mitigate excessive fibrosis [18,38,39]. Thus, rationally designed multifunctional nanomaterials that integrate controlled drug delivery, bioactive functionality, and microenvironment-responsive behavior hold great potential for achieving skin regeneration and developing next-generation regenerative wound therapies. Figure 2 illustrates numerous nanomaterials that can help in the modulation of various signaling pathways that can be impaired in the wound healing process.

## 4. Types of Nanomaterials Used in Wound Healing

The physicochemical and biological properties of various types of nanomaterials have been investigated to facilitate skin regeneration, including metallic nanoparticles (e.g., silver, gold, zinc oxide) with strong antibacterial and anti-inflammatory activities to inhibit infection and control wound microenvironments [40,41], polymeric nanoparticles and nanogels to deliver therapeutic agents (e.g., growth factors, anti-inflammatory drugs, nucleic acids) in a controlled and targeted manner, and nanofibers and nanocomposite hydrogels to mimic the structure of the extracellular matrix and promote cell adhesion, proliferation, and tissue remodeling, which collectively modulate the regenerative pathways and support balanced collagen deposition to prevent pathological scar formation [42].

### 4.1. Metal and Metal Oxide Nanoparticles

Metal and metal oxide nanoparticles are being investigated for wound-healing applications due to their potent antibacterial, anti-inflammatory, and regenerative properties [43,44] including antimicrobial activity, modulation of the inflammatory response, induction of angiogenesis and fibroblast proliferation, and promotion of collagen synthesis and cellular migration. As a result, metal and metal oxide nanoparticles are being studied for use in advanced wound dressings and nanocomposite regenerative systems [45]. Table 1 shows metal-based nanomaterials for advanced wound healing.

The therapeutic activity of metal and metal oxide nanoparticles in wound healing is closely governed by their physicochemical characteristics, including particle size, morphology, surface charge, oxidation state, ion-release kinetics, surface functionalization, and redox activity. These properties determine nanoparticle–protein interactions, cellular uptake, intracellular localization, and interactions with macrophages, keratinocytes, fibroblasts, and endothelial cells, thereby influencing downstream molecular responses. For example, the controlled generation or scavenging of reactive oxygen species (ROS) by metal-based nanoparticles can alter the cellular redox state and subsequently regulate redox-sensitive transcription factors such as NF-κB and Nrf2 [43]. Appropriate suppression of excessive NF-κB activity can reduce persistent production of pro-inflammatory mediators, whereas preservation of physiological inflammatory signaling remains necessary during the early phase of repair. Similarly, nanoparticle-mediated regulation of PI3K/Akt and MAPK/ERK signaling can influence cell survival, proliferation, migration, and cytoskeletal reorganization, thereby supporting re-epithelialization and tissue formation. Regulation of VEGF-associated signaling further contributes to endothelial cell activation and angiogenesis, while modulation of TGF-β/Smad signaling affects fibroblast activation, myofibroblast differentiation, collagen deposition, and extracellular matrix remodeling [46]. Importantly, these effects are highly dependent on nanoparticle dose and ion-release behavior because excessive ROS generation or prolonged pathway activation may produce oxidative damage, cytotoxicity, or pathological inflammation. Thus, the wound-healing effects of metal and metal oxide nanoparticles should be interpreted as the outcome of coordinated regulation of redox balance, inflammatory signaling, proliferative pathways, angiogenic responses, and matrix remodeling rather than as an isolated antimicrobial or wound-closure effect [47].

#### 4.1.1. Silver Nanoparticles

Among the most widely researched nanomaterials for wound healing are silver nanoparticles (AgNPs), which have shown strong antimicrobial, anti-inflammatory, and regenerative properties [48,49]. AgNPs have broad-spectrum antibacterial activity against both Gram-positive and Gram-negative bacteria through membrane disruption, interaction with thiol-containing proteins, and release of Ag^+^ ions that inhibit microbial DNA replication and cellular metabolism [50,51]. AgNPs modulate several critical signaling pathways that are important in wound repair at the molecular level [49]. For example, AgNPs have been associated with attenuation of NF-κB-related inflammatory responses, as reflected by reduced expression of pro-inflammatory cytokines such as TNF-α, IL-1β, and IL-6 [52]. In one study, Ambrožová et al. demonstrated in an in vitro human dermal fibroblast wound-healing model that low concentrations of AgNPs modulated oxidative and inflammatory responses, including ROS generation, Nrf2/NF-κB-related signaling, and IL-6 expression, while also improving fibroblast wound closure, supporting their role in regulating the inflammatory and oxidative microenvironment during wound repair.

AgNPs also stimulate angiogenesis and tissue regeneration by modulating VEGF-associated [53] responses and promoting neovascularization of the wound bed [54]. In a full-thickness wound model, Liu et al. demonstrated that AgNP treatment enhanced wound closure and promoted keratinocyte proliferation and migration, while also stimulating fibroblast-to-myofibroblast differentiation, indicating a direct contribution of AgNPs to the proliferative and tissue-remodeling phases of wound healing [55]. Furthermore, AgNP-containing nanofibers have been shown to regulate the TGF-β1/Smad signaling pathway. Research has demonstrated increased expression of TGF-β1, TGFβRI, TGFβRII, phosphorylated Smad2/3, and collagen I and III following treatment, whereas inhibition of TGF-β receptor signaling using SB431542 attenuated these effects, providing pathway-specific evidence that TGF-β1/Smad signaling contributes to the wound-healing and collagen-regenerative activity of the AgNP-based nanofiber system [56]. AgNPs may also regulate reactive oxygen species (ROS) levels, thereby supporting cellular migration and keratinocyte proliferation during re-epithelialization [48,49]. In an in vivo zebrafish skin-wound model, AgNP treatment was reported to accelerate wound closure and reduce the expression of inflammatory mediators including IL-1β and TNF-α, together with modulation of matrix metalloproteinases and antioxidant responses, further supporting the ability of AgNPs to influence inflammation, oxidative stress, and extracellular matrix remodeling during wound repair [57]. Collectively, these findings indicate that AgNPs promote wound healing through a combination of broad-spectrum antimicrobial activity and modulation of inflammatory, oxidative, proliferative, angiogenic, and extracellular-matrix-remodeling processes. However, the involvement of individual signaling pathways, particularly HIF-1α/VEGF signaling, appears to depend on AgNP formulation, concentration, physicochemical characteristics, and biological context; therefore, these pathways should be considered formulation- and context-dependent rather than universal mechanisms of AgNP-mediated wound healing. In a research study, Aldakheel et al. prepared a green tea leaf extract (GTE)-synthesized AgNP-loaded chitosan-grafted PVA hydrogel via a microwave-assisted method. The presence of AgNPs in the hydrogel matrix was verified by UV–visible spectroscopy, TEM, and FT-IR, and slow release of silver ions facilitated the persistent antibacterial activity. The formulation showed antibacterial activity against Escherichia coli and Staphylococcus aureus in an agar diffusion assay and stimulated the activation and proliferation of fibroblasts, suggesting antimicrobial activity and stimulation of functions important for wound healing. This, however, showed only antimicrobial and in vitro cellular effects and should not be interpreted as complete tissue regeneration or as occurring without scarring. Furthermore, the antibacterial results were achieved under particular experimental conditions; the level of activity may be influenced by the characteristics of the AgNPs, release of Ag+ ions, dose, microbial strain, and assay conditions. More in vivo wound models with adequate follow-up and tissue remodeling and scar-related measures are necessary to confirm the regenerative and translation potential of this hydrogel [58]. In another study, Chinnasamy and co. reduced and stabilized silver nanoparticles using extract from Azadirachta indica (neem) leaves and formulated them into an in situ-forming PF127 hydrogel for topical wound application. The nanoparticles had a spherical shape with an average diameter of about 33 nm and had antioxidant activity, showing a wide antibacterial activity against *Bacillus cereus*, *Escherichia coli*, *Pseudomonas aeruginosa*, and *Staphylococcus aureus*, with SEM observations indicating disruption of the cell wall and cell membrane of the bacteria. The enhanced antibacterial activity, biocompatibility, and the absence of skin irritation further supported the topical applicability of the PF127 hydrogel. The AI-AgNPs-PF127 hydrogel significantly enhanced wound contraction in a mouse wound model. The greater wound closure should be interpreted as wound healing, not as tissue regeneration; however, mice have significant wound contraction, which also plays a role in wound healing. The observed antibacterial, antioxidant, and wound closure activity suggest that the compound has therapeutic applications, but the study failed to demonstrate scar-free regeneration or long-term normal skin architecture. To confirm and translate regenerative success and to assess the aspects of collagen organisation, dermal remodeling, appendage restoration, mechanical properties, and validated scar-related outcomes, further studies with longer follow-up are required [59]. In another study, Mehwish et al. created a silver nanoparticle nanocomposite with polysaccharide extracted from Moringa oleifera seeds (MOS-PS) as a reducing and stabilizing agent. The spectroscopic, microscopic, and physicochemical characterization of the resulting MOS-PS-AgNPs revealed high biocompatibility and low cytotoxicity, and they promoted the migration of fibroblasts in vitro. The nanocomposite also demonstrated antimicrobial activity against wound-associated bacteria, and wound application in a wound model showed improvement in wound contraction, which was correlated with tissue repair in vivo. Histological and RT-PCR data corroborated the biological interpretation, moving beyond wound closure, demonstrating cellular proliferation and tissue remodeling at the wound site. However, this additional wound contraction is not in itself proof of complete regeneration or of wound healing without scarring. The level of regeneration benefit needs to be read in the context of animal models, length of follow-up, and molecular and histological endpoints measured. To support true tissue regeneration and translate these findings to human wounds, longer-term assessments of collagen architecture, dermal appendage restoration, myofibroblast persistence, mechanical properties, and validated scar outcomes would be beneficial [60]. In another study, Qubtia et al. used taro corm extract as a green synthesis method to synthesize silver nanoparticles (AgNPs). UV–visible spectroscopy confirmed the formation of TCE-AgNPs, while FTIR, SEM, EDX, DLS, and XRD analyses resulted in crystalline, spherical nanoparticles with an average size of 244.9–272.2 nm, PDI of 0.530, and a zeta potential of −18.8 mV. The nanoparticles exhibited significant antibacterial effects against several pathogenic bacteria, namely Cronobacter sakazakii, Pseudomonas aeruginosa, Listeria monocytogenes, and Enterococcus faecalis, as well as antioxidant and free-radical scavenging effects. The antimicrobial and antioxidant effects of TCE-AgNPs could be beneficial for wound-management applications, as they may help lower microbial levels and oxidative stress in wounds. If the reported wound healing properties are largely based on antimicrobial, antioxidant, or wound closure results, however, one should be cautious in the interpretation of these results since they alone are not proof of complete tissue regeneration or scar-free healing. More detailed studies in suitable in vivo wound models with longer time frames and structural and functional endpoints like collagen organization, re-epithelialization, dermal remodeling, scar formation, and mechanical recovery would be necessary to validate their regenerative activity and translational potential [61]. Bold et al. produced a burn wound ointment with biologically synthesized silver nanoparticles (AgNPs) by using Rhodiola rosea and sheep tail ointment in another study. The AgNPs were found to have high colloidal stability (zeta potential –68.38 ± 3.4 mV), high crystallite size (about 23 nm), average grain size (67.5 nm), and a crystalline, spherical morphology. The introduced antibacterial activity of the nanoparticles was observed against both Gram-positive and Gram-negative bacteria, and the dose-dependent antioxidant activity of the AgNP-loaded ointment was observed. In BALB/c mice, treatment with the preparation shortened wound healing time, decreased wound size and epidermal thickness, and reduced the migration of mast cells compared to untreated burn wounds. Importantly, a modulation of the gene expression of both pro- and anti-inflammatory genes gives further biological evidence for the effect on the inflammatory phase of wound repair. The rapid wound closure seen in mice does not necessarily equate to complete tissue remodeling or scar-free healing, however, because wound contraction makes a significant contribution to wound closure in mice. Furthermore, while changes in gene expression and histological analyses indicate enhanced tissue repair, further studies are necessary to determine true regenerative efficacy, including collagen architecture, dermal remodeling, restoration of appendages, mechanical properties, and validated scar outcomes. Hence, the results suggest that the ointment containing AgNPs might be a promising formulation for enhancing burn-wound healing, which needs to be evaluated in clinically relevant models and extended follow-up [62]. Aassar et al. created an electrospun nanofibrous wound dressing with hyaluronic acid and polygalacturonic acid (PGA) containing AgNPs that were synthesized using green extracts. The formation and incorporation of the AgNPs were confirmed by UV–visible spectroscopy and TEM, and the fabricated nanofibers showed good wettability and physicochemical properties suitable for wound-dressing applications. The antimicrobial activity of nanofibers loaded with AgNPs was found to be effective against both Gram-positive and Gram-negative bacteria, suggesting their ability to significantly decrease the microbial count at the wound site. However, the results of that study mostly indicate the suitability and antimicrobial activity of the dressing and do not prove its effectiveness in improving tissue regeneration or reparative healing without scars. The dressing is yet to be evaluated in terms of regeneration potential in terms of wound closure, architecture, collagen organization, dermal remodeling, and scar-related outcomes. Future in vivo testing in clinically relevant wound models and with appropriate follow-up is therefore needed to establish whether the antimicrobial action results in better wound healing and functional tissue restoration [63]. In another study, iturin-AgNPs were encapsulated in a chitosan (CS) composite sponge and successfully developed as a low-toxicity wound-dressing material, with high porosity and water-absorption capacity that facilitates the management of exudate in wound healing and keeps the wound moist. The CS sponge was more effective in inhibiting bacterial growth than commercial dressings containing AgNPs at low AgNP doses, and in vitro assays revealed the ability of the CS sponge to inhibit bacterial growth effectively. In an in vivo wound model, the iturin-AgNP-loaded CS dressing demonstrated decreased bacterial infections, increased collagen deposition, more re-epithelialization, and faster wound closure. These results suggest that a synergistic action of both antimicrobial and tissue-repair activities has a positive influence on wound healing, but it is neither scar-free regeneration nor restoration of normal tissue architecture. Moreover, the benefits of AgNP dressings compared to conventional dressings should be considered under specific experimental conditions, as the efficacy of AgNPs against the bacteria could differ depending on the concentration of the AgNP, the properties of the nanoparticles, the release of silver ions, the bacterial strain, and the dressing composition. Studies of collagen organization, dermal remodeling, and restoration of the appendages, and evaluation of mechanical properties and validated scar outcomes over longer periods of time would generate greater evidence for true tissue regeneration and clinical translation [64]. Gawad et al. modified the properties of a bioinspired chitosan–collagen-based hydrogel by using genipin as a crosslinking agent and loading it with AgNPs prepared from folic acid as a reducing agent, followed by loading with cefotaxime sodium. The optimized hydrogel had high swelling capacity and porosity, allowing moisture retention and nutrient exchange, and the system containing AgNPs was found to be effective against both Gram-positive and Gram-negative bacteria. In an injured rat model, the formulation resulted in more than 98% wound contraction and total wound closure within 2–3 weeks, along with decreased malondialdehyde (MDA) levels and increased superoxide dismutase (SOD) activity, which showed a reduction in oxidative stress. The results suggest that the formulation can be used for improved wound healing due to its antimicrobial and antioxidant properties; however, the high percentage of wound contraction and complete closure in rats should not be interpreted as definitive evidence for complete tissue regeneration, as wound contraction makes a significant contribution to wound closure in rodents. Moreover, while lower levels of oxidative stress and better scar closure would be good indicators of healing, long-term structural regeneration would need to be evaluated by studying collagen organization, dermal remodeling, appendage restoration, mechanical properties, and validated scar outcomes. Thus, the regenerative properties of these claims should be viewed in the context of the limitations of the rat wound model and the time period under observation, and other studies in clinically relevant models would need to be conducted to determine the potential for translation [65]. Pansara et al. formulated chitosan-stabilized AgNP-incorporated chitosan film (CH–AgNP–CHF) possessing optimum physicochemical properties and having high antibacterial efficacy against Escherichia coli. The formulation was found to provide a wound closure rate of 3–21 days in vivo, which was faster than marketed SilverKind^®^ Nanofine gel, blank chitosan film, and gauze. The results suggest better wound healing and antimicrobial activity, but wound closure in animals should not be viewed as a measure of the extent of tissue repair, since wound contraction plays a significant role in wound healing in animals. Furthermore, the improvements seen under experimental conditions should not be extrapolated to any other silver-based dressings. To confirm the regenerative efficacy, long-term assessment of tissue remodeling and scar-related outcomes would be necessary [66]. On a wider scale, silver nanoparticle-based systems can be designed to co-deliver several therapeutic agents, including antibiotics, anti-inflammatory drugs, growth factors, and natural antioxidants, to obtain a synergistic wound healing effect. The incorporation of AgNPs into advanced platforms such as hydrogels, nanofibers, films, and sponges for the controlled release of drugs, retention at the wound site, and penetration into infected tissues is also being investigated. Future studies should aim to develop stimuli-responsive and multifunctional AgNP-based nanocomposites that can dynamically modulate inflammation, oxidative stress, and microbial load; the combination of AgNPs with biopolymers or bioactive molecules can enhance biocompatibility and reduce cytotoxicity.

#### 4.1.2. Gold Nanoparticles

Gold nanoparticles (AuNPs) are multifunctional in wound healing because they exhibit both antimicrobial activity and the ability to modulate key cellular signaling pathways [67]. Their antimicrobial effect is due to their interaction with bacterial cell membranes, resulting in disruption of membrane integrity, increased permeability, and induction of oxidative stress, which inhibits bacterial growth and biofilm formation [68]. AuNPs can also increase the efficiency of traditional antibiotics. AuNPs modulate major signaling pathways for tissue repair, including suppression of the NF-κB pathway to inhibit inflammation and production of pro-inflammatory cytokines [69], activation of the PI3K/Akt pathway to promote cell survival, proliferation, and migration, and stimulation of the MAPK/ERK pathway to promote cellular growth and VEGF-mediated angiogenesis to form new blood vessels. These mechanisms promote wound healing by inhibiting infection and facilitating efficient tissue regeneration through synergistic action against both Gram-positive and Gram-negative bacteria [70]. In one study, Mahmoud et al. compared the efficiency of AuNP-loaded thermosensitive AuNP-P407 hydrogels of various shapes and surface properties. After 14 days, PEGylated and cationic Au nanorod formulations exhibited higher antibacterial activity and better wound-healing properties, such as re-epithelialization and collagen deposition, as well as being able to modulate inflammatory and anti-inflammatory gene expression. The results indicate that surface properties of the nanoparticles might affect the biological response, but the enhancement in the wound healing process should not be considered complete tissue regeneration and scar-free healing, as the study was conducted after 14 days. Additional long-term investigations examining collagen organization, dermal remodeling, appendage restoration, mechanical properties, and scar outcomes need to be performed to confirm regenerative efficacy and clinical relevance [71]. In another study, Arafa and colleagues created AuNP-containing thermoresponsive gels made of Pluronic^®^ F127 alone (F1) or co-combined with hydroxypropyl methylcellulose (F2). The two formulations showed physiological-range gelation properties, with F2 showing higher bioadhesion and gel strength properties that may lead to better retention at the wound level. The formulations exhibited antibacterial activity against *S. aureus* and in vivo wound healing potential, with histopathological results corroborating better tissue repair. These results are preliminary and are not conclusive evidence for scar-free tissue regeneration; however, without long-term structural and functional evaluation, these results should be interpreted as wound healing. The reported differences between F1 and F2 also show the effect of the formulation composition on biological performance, and thus, the formulation’s efficacy cannot be generalized and is specifically related to the incorporation of AuNP as the only component. More long-term studies are needed to demonstrate the real regenerative effectiveness and translatability of collagen organization, dermal remodeling, appendage restoration, and scar-related outcomes [72]. In another study, the authors reported a green, sunlight-assisted, hydrogel-based synthesis of gold nanoparticles (AuNPs) using seed-derived hydrogel from *Cydonia oblonga* as a reducing and stabilizing agent. The AuNPs synthesized had unique morphologies (cubic and rectangular), with an average size of about 74 nm and a surface plasmon resonance at 560 nm, and they exhibited significant antimicrobial activity with clear inhibition zones and up to 95% suppression of growth at a low concentration (16 µg/mL) after 24 h. The in vivo murine wound models also indicated rapid, efficient healing with nearly complete (99%) wound closure within 5 days, and molecular analysis revealed upregulation of the regenerative markers NANOG and CD-34, suggesting stimulation of cell proliferation and angiogenesis [73]. Li et al. designed a porous chitosan dressing in which Au–Ag bimetallic nanoparticles (~10 nm) were evenly distributed, which accelerated the release of silver ions and the antibacterial activity, and simultaneously reduced the cytotoxicity and improved the swelling, moisture retention, and mechanical properties. The in vivo data demonstrated an ability to heal wounds, which further supports the potential of CS–Au–Ag as an antimicrobial wound management option. Yet, rapid wound closure does not necessarily mean total tissue regeneration or scar-free healing, especially in animals where wound contraction plays a significant role in wound closure. The improvement in performance should also be understood in the context of the composition of the nanoparticles and the release of silver ions, not in the general context of the use of gold. To demonstrate regenerative efficacy and clinical translation, longer-term evaluation of tissue architecture and collagen remodeling, restoration of dermal appendages, mechanical recovery, and scar outcomes would be needed [74]. In another study, combined photobiomodulation (PBM) and gold nanoparticle-linked hyaluronic acid (GNPs-HA) enhanced wound healing in an epithelial lesion model by decreasing pro-inflammatory cytokines and oxidative-stress markers and increasing anti-inflammatory cytokines and growth factors, such as TGF-β and FGF. Histological examination also revealed decreased inflammatory infiltration, increased fibroblast activity and neovascularization, and increased collagen deposition with accelerated wound healing. While this indicates greater control over inflammation and tissue repair, increased expression of TGF-β and deposition of collagen does not equate to scar-free regeneration, and the more rapid rate of closure does not mean the tissue is completely restored. A longer-term collagen architecture assessment as well as dermal remodeling, appendage restoration, mechanical properties, and validation of scar outcomes would be needed to prove true regenerative efficacy [75]. He and colleagues describe the development of a new self-delivery antimicrobial platform involving a combination of polyhexamethylene biguanide (PHMB) and gold nanoparticles (PHMB@Au NPs), free of conventional drug carriers. The combination of the antibacterial and anti-biofilm properties of PHMB and the photothermal effects of gold nanoparticles under near-infrared irradiation showed a synergistic effect and led to efficient bacterial elimination, strong inhibition of biofilm formation, and rapid infection control and wound healing in vivo, accompanied by enhanced tissue regeneration, the polarization of macrophages from the pro-inflammatory M1 type to the anti-inflammatory M2 type, and induction of angiogenesis [76].

Salama et al. prepared AuNPs loaded with curcumin, formulated in Pluronic^®^ F127 gel, to improve permeation, sustain the release of curcumin, and increase antibacterial and antibiofilm activities against both Gram-positive and Gram-negative bacteria. The formulation was found to promote wound healing in a diabetic rat wound model and was correlated with a decrease in oxidative stress, improvement in antioxidant and anti-inflammatory activities, and an increase in collagen deposition. These results indicated that the closure process was enhanced and that collagen deposition resulted in a decrease in inflammation and fibrosis; however, accelerated closure and more collagen deposition do not imply complete tissue regeneration or scar-free healing. The findings should also be viewed in the context of time limits of the diabetic rat model and study duration, and a longer-term evaluation of collagen organisation, dermal remodeling, appendage restoration, mechanical properties, and validated scar outcomes would be required to demonstrate the efficacy of regeneration and the relevance to human use [77]. Kolarijani and co-workers prepared poly(ε-caprolactone)/gelatin nanofibrous scaffolds loaded with AuNPs that exhibited good blood compatibility, biocompatibility, and resistance to microbial penetration while also promoting the growth of fibroblasts (400 ppm formulation). The AuNP-loaded scaffold also promoted wound healing in a full-thickness wound model in rats, which suggests the possibility of using it as a wound-dressing platform. The improvement observed must be taken as wound healing improvement but not as tissue regeneration, especially since wound contraction plays a major part in wound closure in rats. Furthermore, its superior performance is only on the specific experimental comparator and is not proof of superiority over other high-tech wound dressings in general. Further studies of collagen organization, dermal remodeling, appendage regeneration, mechanical recovery, and clinically validated scar outcomes would be necessary to support the regenerative efficacy and clinical translation [78]. In another study, a silk fibroin/collagen composite nanofiber loaded with bimetallic silver and gold nanoparticles was successfully fabricated, and the nanoparticles were found to be about 6.76 nm (Ag) and 9.75 nm (Au), with the composite nanofiber being about 189.46 ± 108.73 nm in diameter. The composite had good antibacterial activity (zones of inhibition of 17 mm for *S. aureus* and 18 mm for *E. coli*)*,* good biocompatibility (based on increased proliferation of NIH3T3 fibroblast cells), good blood compatibility (hemolysis rates of 1.5% to 3.9%), high swelling capacity, efficient absorption of wound exudates, ability to promote cell adhesion and tissue regeneration, and good therapeutic efficacy in vivo (approximately 98.85% of wounds healed in Sprague–Dawley rats) [79]. Taay et al. used the antioxidant activity and biological effects of the extract of *Brassica oleracea* (*B. oleracea*) to synthesize biogenic AuNPs. The AuNPs also accelerated wound contraction, collagen production, and re-epithelialization in a wound model, suggesting their potential in wound healing. However, such results are not sufficient proof of total regeneration or scarless healing, especially if significant wound contraction is involved in the closure of the wound. The results are thus not indicative of a definitive regenerative restoration, but rather of improved wound repair. To confirm tissue regeneration and to assess translation, longer-term evaluation of collagen architecture, dermal remodeling, appendage regeneration, mechanical properties, and validated scar outcomes would be required [80]. The studies discussed here indicate that similar strategies can be applied to other therapeutic agents by loading them into AuNP-based systems to increase stability, targeted delivery, and controlled release at the wound site, and conjugation or encapsulation of drugs like antibiotics, anti-inflammatory agents, growth factors, and natural bioactives (e.g., curcumin) within AuNP-integrated hydrogels, nanofibers, or scaffolds can improve bioavailability and minimize systemic side effects. This can enhance wound healing by combining antimicrobial action, modulation of inflammatory pathways (e.g., NF-κB), promotion of cell proliferation (PI3K/Akt, MAPK/ERK), and stimulation of angiogenesis, while synergistic approaches (e.g., bimetallic nanoparticles or photothermal therapy) can further enhance infection control and tissue regeneration.

#### 4.1.3. Zinc Oxide Nanoparticles

Zinc oxide nanoparticles (ZnO NPs) are novel nanomaterials that have been shown to possess antimicrobial, anti-inflammatory, antioxidant, and pro-regenerative effects, with broad-spectrum antibacterial activity against Gram-positive and Gram-negative bacteria by generating ROS, releasing Zn^2+^ ions, and causing membrane damage, inhibiting infection and biofilm formation [81,82]. Mohsin Rayyif et al. demonstrated that ZnO NP-modified wound dressings exhibited strong antibacterial activity against both Gram-negative and Gram-positive wound pathogens, including Pseudomonas aeruginosa, Escherichia coli, Staphylococcus aureus, and Enterococcus faecalis, while significantly impairing biofilm development for up to 48–72 h, particularly at ZnO NP loadings of 0.6 and 0.9% [83]. ZnO NPs act at the molecular level to regulate major signaling pathways involved in tissue repair, such as the NF-κB pathway to inhibit inflammation, the PI3K/Akt pathway to promote cell survival and proliferation, and the MAPK/ERK and TGF-β pathways to stimulate fibroblast activity, collagen synthesis, and extracellular matrix remodeling [84]. Tang et al. provided direct molecular evidence for the anti-inflammatory activity of ZnO NPs by demonstrating that treatment of LPS-stimulated RAW264.7 macrophages with ZnO NPs reduced TNF-α, IL-1β, IL-6, NO, iNOS, and COX-2 expression. Western blot analysis further showed inhibition of IκB-α degradation and NF-κB p65 nuclear translocation, while ZnO NPs also suppressed JAK1/STAT1/STAT3 activation and reduced intracellular ROS levels, confirming direct modulation of inflammatory signaling [85]. Similarly, Ozdemir et al. reported that ZnO NPs synthesized using Capparis spinosa extract accelerated in vitro wound closure at subcytotoxic concentrations and reduced inflammatory and oxidative stress responses while increasing antioxidant activity. Immunohistochemical analysis further demonstrated increased expression of FGFR2, IGF, and TGF-β, supporting the involvement of growth-factor-mediated signaling in ZnO NP-associated wound repair [86]. Furthermore, ZnO NPs have been reported to stimulate endothelial-cell migration and blood-vessel formation through ROS-mediated activation of the MAPK/Akt/eNOS axis, suggesting that ZnO-associated angiogenesis may involve activation of Akt-dependent endothelial signaling and nitric oxide production [87]. ZnO NPs can be combined with drugs, such as antibiotics, anti-inflammatory agents, growth factors [88], or natural bioactives, within hydrogels, nanofibers, or scaffold systems for controlled and sustained release at the wound site. For example, alginate hydrogels containing ZnO NPs have been developed as wound-healing templates, where ZnO provided Zn^2+^-mediated antimicrobial activity and ROS generation, while the alginate matrix provided a supportive platform for localized treatment [88]. More recently, Yao et al. demonstrated that alginate-modified ZnO NPs markedly improved antibacterial and antibiofilm activity against wound-associated bacteria and accelerated healing of Staphylococcus aureus-infected wounds, with a wound-healing rate of approximately 79.2% compared with approximately 65.8% for unmodified ZnO NPs, illustrating the advantage of surface modification for enhancing the therapeutic performance of ZnO-based wound-healing systems [89]. Collectively, these findings demonstrate that ZnO-based nanoplatforms can combine antimicrobial activity with modulation of inflammatory and oxidative responses, angiogenic signaling, fibroblast activity, and extracellular matrix remodeling, making them promising candidates for advanced wound dressings and regenerative nanomedicine.

ZnO NPs act at the molecular level to regulate major signaling pathways involved in tissue repair, such as the NF-κB pathway to inhibit inflammation, the PI3K/Akt pathway to promote cell survival and proliferation, and the MAPK/ERK and TGF-β pathways to stimulate fibroblast activity, collagen synthesis, and extracellular matrix remodeling. Moreover, ZnO NPs promote angiogenesis by upregulating VEGF and enhance re-epithelialization by stimulating keratinocyte migration and differentiation. ZnO NPs can be combined with drugs, such as antibiotics, anti-inflammatory agents, growth factors, or natural bioactives, within hydrogels, nanofibers, or scaffold systems for controlled and sustained release at the wound site; the combinational approach enhances antimicrobial efficacy while simultaneously modulating inflammation, oxidative stress, and tissue regeneration, making ZnO-based nanoplatforms attractive for next-generation wound healing applications. In one study, Khalid and team showed that ZnO NPs synthesized using leaf extract of Zea mays have great potential as a multifunctional wound-healing agent. The green synthesis method yielded partially spherical, crystalline nanoparticles of nanoscale dimensions, and plant-derived phytochemicals served as reducing and stabilizing agents. The ZnZM NPs exhibited potent antibacterial activity against common wound pathogens, as well as significant antioxidant properties, including free radical scavenging and ferric ion-reducing ability. In vivo evaluation using a topical gel formulation containing 1% ZnZM NPs showed significantly enhanced wound healing compared with controls and standards, with faster wound contraction and superior tissue repair, as confirmed by histopathological analysis. The results indicate that biosynthesized ZnO NPs have great potential in wound healing applications [90]. Khosravian et al. created chitosan patches containing ciprofloxacin and zinc nanoparticles (Cs/Zn@Ci) that yielded accelerated wound closure, better inflammatory response, fibroblast activity, early angiogenesis, re-epithelialization, and collagen organization compared with the control and single-component treatments. Further molecular and biochemical studies revealed not only higher expression of CK14 and EGFR, but also higher expression of collagen and glycosaminoglycans, as well as higher levels of epithelial proliferation and differentiation, providing evidence of tissue repair beyond wound closure alone. The findings of that study, including collagen-related parameters and molecular markers, support the reduction in scar formation claimed in the report, but longer-term, validated scar assessment would be required to make definitive scar-prevention claims. In general, the results indicate better wound healing and tissue remodeling, and additional long-term and clinically relevant studies are warranted to confirm full restoration of regeneration and translate to human wound healing [91]. Ye et al. designed tissue-adhesive PEG hydrogels with polydopamine-functionalized ZnO nanoparticles (PDA@ZnONPs) with tissue adhesion, sustained release of Zn^2+^, antibacterial activity against *S. aureus* and *E. coli*, and antioxidant activity. The hydrogel enhanced in vivo tissue repair and remodeling by promoting anti-inflammatory M2 macrophages and inhibiting pro-inflammatory M1 macrophages, along with enhanced angiogenesis. These results suggest that infected wounds heal better, but closure of the wound and macrophage polarization should not by themselves be regarded as proof of full tissue regeneration and scar-free healing. The effects observed may include immune modulation and enhanced repair of damaged tissue, but direct activation of the pathways is only eligible for claims if there is pathway-specific molecular evidence. Evidence of regenerative efficacy and clinical translation could be strengthened by longer-term assessment of tissue architecture, collagen remodeling, dermal appendage restoration, mechanical recovery, and scar outcomes [92]. Waheed and colleagues showed that ZnBV-NPs can be successfully synthesized using Bauhinia variegata by a green approach and have strong biological activities, as confirmed by characterization techniques, including formation, composition, and functionalization by plant phytochemicals that contribute to their stability. ZnBV-NPs displayed excellent antibacterial activity against major wound-infecting pathogens, as well as high antioxidant potential, which increased with nanoparticle concentration. In vivo evaluation showed that a 1% ZnBV-NPs-loaded carbopol gel markedly improved wound healing, as indicated by increased wound contraction compared to untreated and standard-treated groups. However, cytotoxicity studies showed no significant anticancer activity against HepG2 cells, which demonstrates good biocompatibility [93]. Nandhini et al. synthesized green ZnO nanoparticles using extract of Ocimum americanum and Euphorbia hirta via microwave-assisted synthesis. The nanoparticles exhibited antioxidant, anti-inflammatory, anti-bacterial, and anti-biofilm properties, in addition to lowering the bacterial load and improving the migration and proliferation of fibroblasts in suitable concentrations. The discovery of their potential to improve wound healing, by tackling three aspects of wound healing at the same time, supports their use in wounds. The responses of the fibroblasts and the antimicrobial activity observed, however, do not necessarily mean complete regeneration of tissue or regeneration without scarring, and comparisons with normal drugs must be interpreted in the context of the specific experimental conditions. Additional in vivo studies for sufficient follow-up and to assess tissue remodeling, collagen architecture, production of dermal appendages, mechanical properties, and scar outcomes would be needed to confirm their regenerative efficacy and potential for translational application [94]. Liu et al. synthesized a double network TA-GL/OSA/ZnO composite hydrogel, which exhibited enhanced mechanical stability, antibacterial activity against *S. aureus* and *E. coli*, antioxidant activity, and controlled release of Zn^2+^. In vivo, the hydrogel has been found to support wound healing and accelerate the healing of the epithelium, which shows its potential for the management of infected wounds. However, the results of such studies are only an improvement in wound repair and do not prove true scarless tissue regeneration, because enhanced epithelialization and antibacterial activity alone do not constitute restoration of normal tissue architecture. The role of ZnO and tannic acid could also include formulation composition and Zn2+ release. Further studies of collagen organization, dermal remodeling, restoration of appendages, mechanical properties, and clinically relevant scarring outcomes would be necessary to prove full tissue regeneration and translation [95]. Wang and co. fabricated oxidized sodium alginate (OSA) fiber membranes with the addition of ZnO nanoparticles (NPs). The ZnO-containing membranes showed anti-inflammatory effects and were found to be biocompatible, and they also demonstrated antimicrobial activity against both *E. coli* and *S. aureus*. In vivo, the composite membranes improved epithelialization and neovascularization compared to membranes without ZnO, indicating better wound healing. These results should not be taken as absolute proof of full tissue regeneration, though, because improved epithelialization and neovascularization do not indicate restoration of normal tissue architecture or a lack of scarring over the long term. The benefit of the ZnO-free membrane is only applicable for the experimental model and formulation conditions. Assessment of collagen organization, dermal remodeling, restoration of appendages, mechanical properties, and validated scar outcomes over longer time periods will reinforce the evidence of regenerative efficacy and potential for translation [96]. Shahrousvand et al. designed a PVP–PAA hydrogel loaded with ZnO nanoparticles that served as an antibacterial wound healing gel. The ZnO-loaded hydrogel accelerated wound contraction in a rat excisional wound model, and the histological results confirmed that it promoted better wound repair. The observed healing response may have been due to the antibacterial activity and hydrogel microenvironment. Increased wound contraction in a rat model should not, however, be taken as proof of full tissue regeneration, as there is a significant contribution of wound contraction to wound closure in rats. True regenerative efficacy and relevance to translation require longer-term assessment of collagen organization, dermal remodeling, appendage restoration, mechanical properties, and validated scar outcomes [97]. These studies demonstrate that these tiny particles (such as zinc oxide nanoparticles) can be like smart helpers for wound healing, killing bacteria (preventing infection), reducing swelling, and speeding up the body’s repair of damaged skin. If these nanoparticles are embedded in materials such as gels, patches, or fibers, they remain on the wound and gradually release their effect, making healing more efficient; adding medicines such as antibiotics or natural compounds to these systems further enhances their activity by simultaneously fighting infection and accelerating tissue growth. As a result, these new advanced wound dressings are more effective than traditional dressings because they protect the wound, manage infection, and actively promote the body’s efforts to regenerate healthy skin.

#### 4.1.4. Copper-Based Nanomaterials

Copper-based nanomaterials have been used in advanced wound healing [98] because they can simultaneously regulate several cellular and molecular signaling pathways; after application, copper-based nanomaterials release Cu^2+^ ions that can activate the HIF-1α/VEGF pathway to promote angiogenesis and oxygen and nutrient supply, the TGF-β/Smad pathway to promote fibroblast proliferation, extracellular matrix remodeling, and collagen (especially type I) synthesis, and the MAPK and PI3K/Akt pathways to support keratinocyte migration, cell survival, and rapid re-epithelialization, and they can induce moderate amounts of ROS, which can serve as secondary messengers that enhance cell signaling and exert strong antibacterial effects by damaging microbial membranes, proteins, and DNA [99]. Borkow et al. demonstrated that copper oxide-impregnated dressings increased HIF-1α and VEGF expression, enhanced blood-vessel formation, and accelerated wound closure in diabetic mice compared with control treatment [100]. Gopalakrishnan et al. further reported that CuNPs promoted skin-cell migration and proliferation, neovascularization, granulation-tissue formation, and rapid closure of full-thickness wounds, with 80-nm CuNPs at 1 μM showing pronounced regenerative activity [101]. Additionally, copper-dependent enzymes such as lysyl oxidase also contribute to collagen cross-linking and tissue strength [102]. Copper-based nanomaterials have demonstrated promising antimicrobial activity against a range of Gram-positive and Gram-negative pathogens. However, their antimicrobial efficacy and biocompatibility are highly dependent on factors such as nanoparticle composition, size, surface chemistry, dose, ion release kinetics, microbial strain, and experimental conditions. Therefore, copper-based nanomaterials should be considered promising alternatives rather than universally superior to silver-based nanomaterials [103] (which can become cytotoxic at higher concentrations), while remaining biocompatible at controlled doses, being more cost-effective, and having a direct physiological role in tissue repair without the need for additional growth factors or additives, making them superior candidates for next-generation wound healing therapies. Tahvilian et al. prepared green-synthesized copper nanoparticles using the extract of Allium saralicum, which showed high antioxidant activity, broad-spectrum antibacterial and antifungal activities, and good cytocompatibility. In vivo, application of CuNP-containing ointment resulted in faster wound closure, as well as better tissue repair parameters such as decreased inflammatory-cell infiltration, higher hydroxyproline and hexosamine content, and increased fibroblast activity. While these results suggest enhanced wound healing, wound contraction in animal models does not necessarily indicate full tissue regeneration. Furthermore, the antimicrobial properties of CuNPs cannot be considered superior to silver-based nanomaterials, but they must be evaluated with regard to certain particle characteristics, amounts, microorganism type, and experimental conditions [104]. Zhao et al. synthesized CuNPs using the leaf extract of Allium eriophyllum, which exhibited antibacterial and antifungal activity at relatively low concentrations and wound-healing benefits in animal models. Treatment resulted in accelerated wound contraction, increased hydroxyproline and hexosamine levels, increased fibroblast activity and angiogenesis, and decreased wound size and inflammatory-cell infiltration. The results suggest the wound healing process and antimicrobial activity but do not confirm complete wound regeneration based on measurements of wound contraction and biochemical markers. Moreover, the antimicrobial activity reported should not be considered as being superior to conventional antibiotics or silver-based nanomaterials, but should be understood as being tested with regard to specific doses, microbial strains, and particle characteristics under experimental conditions [105]. In another study, a new copper-based polymer metal–organic framework loaded with silver nanoparticles was developed as an effective antibacterial system for wound healing. The material had a nanosheet-like structure with a large surface area and porosity, which enabled the loading and slow release of silver ions and minimized the release of copper ions, resulting in improved biocompatibility and reduced haemolysis. The in vitro experiments demonstrated that the hybrid material efficiently killed bacteria by damaging their cell membranes and metabolic functions through the generation of reactive oxygen species. In vivo studies showed that the material significantly promoted the healing of infected wounds by enhancing tissue regeneration and stimulating the formation of dense collagen. The results suggest that this polymer–MOF-based hybrid is a promising and efficient platform for antibacterial wound treatment, with enhanced safety and therapeutic performance [106]. Pourshahrestani et al. synthesized tannic acid (TA)-loaded copper- and zinc-doped mesoporous bioactive glass nanoparticles (CuMBGNs-TA and ZnMBGNs-TA) that exhibited antioxidant activity, excellent biocompatibility with fibroblasts, and hemocompatibility. Under the same experimental conditions, ZnMBGNs-TA was found to result in wound closure of over 90%, while CuMBGNs-TA resulted in 66–83% wound closure. The results showed that the wound closure obtained after treatment with ZnMBGNs-TA was more than 90%, whereas the wound closure obtained after treatment with CuMBGNs-TA was 66–83%. These in vitro wound closure results are not to be considered definitive proof of tissue regeneration, and the superiority in wound closure seen with ZnMBGNs-TA compared to the other products does not demonstrate general superiority due to formulation composition and experimental conditions. Additional in vivo and long-term studies are required to evaluate the regenerative properties and scar-related effects [107]. In another study, Ali et al. successfully synthesized copper oxide nanoparticles (CuO-NPs) via a scalable and environmentally friendly chemical synthesis method, with key parameters optimized for producing a stable colloidal system for biomedical applications. The CuO-NPs were characterized and showed good antibacterial activity against multidrug-resistant pathogens associated with diabetic wounds, such as *Pseudomonas aeruginosa*, *Proteus vulgaris*, and methicillin-resistant *Staphylococcus aureus*. The developed nanoparticles were found to be biocompatible, with no skin irritation in rat models, and in vivo studies showed that CuO-NPs greatly enhanced wound healing in diabetic conditions, with complete healing of non-infected and infected wounds at days 15 and 18, respectively, substantially faster than a commercial wound dressing, at a lower dose than similar nanoparticles [108]. Cai et al. designed an electrospun nanofiber membrane with silk fibroin-templated CuS nanoparticles, which exhibited excellent cytocompatibility, hemocompatibility, and NIR-responsive photothermal antibacterial properties. The dressing decreased IL-6 and increased VEGF in infected wounds, leading to quicker healing and increased collagen deposition and neovascularization. These results provide a rational basis for better healing in infected wounds because of antimicrobial, anti-inflammatory, and pro-angiogenic properties; however, these results alone do not indicate scar-free regeneration. Therefore, the effects must be seen in the context of the specific photothermal conditions and the type of wound, and further studies need to be conducted to evaluate the regenerative effects with regard to long-term collagen organization, tissue architecture, and scar outcomes [109].

Li et al. synthesized an injectable, self-healing, and adhesive photothermal hydrogel (mCS-Cu-Ser1) using gallic acid-grafted chitosan and copper-mediated cross-linking, as well as sericin nanoparticles. The hydrogel temperature rapidly increased to ~50 °C under NIR irradiation and was found to have strong antibacterial activity against MRSA, in addition to its ROS-scavenging capacity, which was found to decrease oxidative stress. These synergisms had a positive impact on wound healing in infected wounds and reinforced the idea that it has the capability to be developed as an alternative platform that is not dependent on antibiotics. The therapeutic effect, however, should be used in the context of the specific photothermal conditions and wound model, and the healing effects should not be considered as full tissue regeneration, but should be assessed over a long period, which enables the evaluation of tissue remodeling and scar outcome [110]. These formulations, which combine the biological activity of copper with advanced delivery systems such as nanoparticles, hydrogels, and nanofibers, enhance wound healing by promoting angiogenesis, collagen deposition, and cell proliferation while simultaneously providing strong antibacterial effects against drug-resistant strains, producing mild reactive oxygen species that further improve microbial killing and cellular signaling for tissue repair, incorporating bioactive agents such as tannic acid, zinc, or plant extracts to enhance antioxidant and anti-inflammatory responses, maintaining a moist environment and allowing sustained release and improved tissue adhesion via platforms like hydrogels and electrospun membranes, ultimately leading to faster wound closure and decreased infection, as well as greater tissue regeneration.

**Table 1:** Current status of research on metal-based nanomaterials for advanced wound healing.

S. No.	Nanomaterial	Physicochemical Characteristics	Therapeutic Cargo	Wound Model	Safety Assessment	Mechanism in Combating Wound Healing	References
**1**	*Scutellaria barbata*-AgNPs/cotton fabric	Nano-sized AgNPs; characterized by TEM, AFM, FTIR, and XRD	*S. barbata* phytochemicals	L929 fibroblast scratch assay	MTT assay showed favorable L929 cell viability under tested conditions	Silver nanoparticles synthesized using *Scutellaria barbata* extract showed significant antimicrobial and antibiofilm activity against bacterial and fungal strains, with effective performance when incorporated onto cotton fabric. The nanoparticles were non-toxic to L929 fibroblasts and enhanced wound closure in scratch assays, supporting their potential for improved wound management without implying complete tissue regeneration.	[111]
**2**	AgNP-loaded collagen nanofibers	Fiber diameter 300–700 nm; sustained Ag-ion release	AgNPs	Animal wound model; *S. aureus*/*P. aeruginosa* antimicrobial testing	Biodegradable collagen scaffold; no adverse safety outcome highlighted	Rath and colleagues developed AgNP-loaded collagen nanofiber mats with sustained silver-ion release and strong antimicrobial activity against *S. aureus* and *P. aeruginosa*. In vivo, the mats accelerated wound closure with enhanced re-epithelialization and collagen deposition compared with pure collagen mats, supporting improved wound healing.	[112]
**3**	Plant-mediated AgNPs	Characteristic UV–Vis absorption at 450 nm; spherical AgNPs	Plant-derived phytochemicals	Fibroblast wound-healing assay	Cytotoxic/apoptotic effects were formulation- and condition-dependent	Ahn and co-workers synthesized spherical AgNPs with a characteristic absorption peak at 450 nm and strong antioxidant activity, while *Lindera strychnifolia*-derived AgNPs showed wound-healing potential in fibroblast assays. However, their biological activity was influenced by serum conditions, highlighting the importance of experimental context when interpreting nanoparticle effects.	[113]
**4**	PCL/Gel/AgNP nanofibrous membrane	Plasma-treated PCL; gelatin–AgNP multilayer coating	AgNPs	Mouse wound model	Reduced wound-scaffold adhesion may minimize tissue damage during dressing removal	Plasma-treated PCL scaffolds coated with gelatin-embedded AgNPs showed enhanced hydrophilicity, absorptive capacity, and coating thickness, improving interaction with the wound environment. The EsPCLGelAg membrane also exhibited reduced adhesion to the wound site, minimizing tissue damage during dressing removal.	[114]
**5**	Crosslinked alginate/AgNP film	p-Phthaloyl-crosslinked alginate	AgNPs + imidazolium ionic liquid	In vivo wound model	Antibacterial activity demonstrated; detailed long-term toxicity not reported	The p-phthaloyl-crosslinked alginate/AgNP film showed antibacterial activity, with inhibition zones of 10 mm for the AgNP film and 23 mm for the ionic-liquid film. In vivo, the AgNP film achieved 97% wound closure by day 7, compared with 93.25% by day 9 for the ionic-liquid film.	[115]
**6**	Silk fibroin/AuNP 3D nanofibrous matrix	~24 nm AuNPs; 3D electrospun matrix	AuNPs	In vitro and animal wound model	Maintained biocompatibility in reported experiments	The 3D silk fibroin/AuNP nanofibrous matrices improved mechanical strength, cell spreading, neovascularization, and granulation tissue formation while maintaining biocompatibility. In vitro and in vivo, AuNP incorporation produced slightly enhanced wound healing compared with controls.	[116]
**7**	CS/PVA/AuNP film	Spherical AuNPs, 20 nm	AuNPs	Antibacterial model; wound-healing application	Not reported in detail	CS/PVA films containing 20 nm spherical AuNPs showed enhanced crystallinity and strong visible-light antibacterial activity. The inhibition zones increased from 4.2 to 13.1 mm against *E. coli* and 6.4 to 24.8 mm against *S. aureus* with increasing AuNP loading.	[117]
**8**	Collagen-I@AuNPs	AuNPs 19 ± 0.2 nm	AuNPs stabilized with collagen-I	Human skin fibroblast scratch model	No apparent cytotoxicity; cellular uptake demonstrated	Collagen-I@AuNPs reduced IL-6 and TNF-α while increasing bFGF and VEGF expression in human skin fibroblasts, supporting anti-inflammatory activity and angiogenesis. The treatment promoted faster wound closure without cytotoxicity, indicating good biocompatibility and wound-healing potential.	[118]
**9**	Os/Au-PDA@PLGA composite membrane	PLGA-based electroactive composite	AuNPs + antibacterial peptides	In vivo wound model	Reported biocompatibility and hemocompatibility	The Os/Au-PDA@PLGA composite membrane showed enhanced antibacterial, antioxidant, electrical, and hydrophilic properties, promoting cell proliferation and migration. In vivo, it improved collagen deposition and vascularization, while electrical stimulation further accelerated wound healing compared with pure polymer.	[119]
**10**	Ultrasmall AuNP/CMCS hydrogel	AuNPs 1–3 nm; self-healing hydrogel	AuNPs	In vivo wound model	Good reported biocompatibility	PAA-CMCS-Au hydrogel containing 1–3 nm ultrasmall AuNPs showed enhanced antibacterial activity through interaction with negatively charged bacterial cells while maintaining good biocompatibility and self-healing properties. In vivo, the structurally stable hydrogel supported effective wound healing.	[120]
**11**	*Woodfordia fruticosa* AuNPs	AuNPs 13 ± 1.2 nm	Plant-derived phytochemicals	Wistar rat wound model	Animal study supported tolerability; detailed systemic safety not established	Microneedle-based drug delivery enables minimally invasive and localized drug delivery to ocular tissues, improving drug retention and bioavailability while reducing dosing frequency and systemic exposure. Its painless application may also improve patient compliance and therapeutic efficacy in ocular disease management.	[121]
**12**	Aloe-mediated ZnO-NPs/silica-gel dressing	ZnO-NPs ~44 nm	Plant phytochemicals	Mouse wound model	No major adverse outcome reported	Green-synthesized ZnO-NPs using *Aloe barbadensis* extract showed 90–92% adsorption efficiency for Malachite green and Congo red, along with broad-spectrum antimicrobial activity. ZnO-NP/silica-gel dressings promoted faster tissue repair in mice compared with controls, supporting their potential for wound management.	[122]
**13**	*Calendula officinalis*-ZnO NPs	17.66 nm; UV–Vis peak 355 nm	Plant-derived phytochemicals	Cell migration/wound-closure assay	Low cytotoxicity in reported assay	Green-synthesized ZnO-NPs using *Calendula officinalis* extract had an average size of 17.66 nm and a UV–Vis peak at 355 nm, with low cytotoxicity, moderate antioxidant activity, and enhanced cell migration. The nanoparticles also improved wound closure compared with the control, supporting their potential for antioxidant-assisted wound healing.	[123]
**14**	*Ocimum sanctum*-ZnO NPs	40–70 nm; stable/agglomerated morphology	Plant-derived phytochemicals	Antibacterial/antibiofilm model	Stability and residual phytochemical profile assessed; detailed cytotoxicity not established	Green-synthesized ZnO-NPs from *Ocimum sanctum* leaf extract showed potent antibacterial and antibiofilm activity against *P. aeruginosa*, with higher efficacy than streptomycin. The nanoparticles also exhibited good stability and sustainable antimicrobial potential for biomedical applications.	[124]
**15**	*Nigella sativa*-ZnO NPs	~45 nm, polydisperse	Plant-derived phytochemicals	Diabetic animal model; metabolic assessment	Animal efficacy reported; dedicated wound-safety assessment limited	Green-synthesized ZnO-NPs from *Nigella sativa* leaf extract had an average size of **~45 nm** and showed antidiabetic effects by improving glycogen storage, insulin levels, and blood glucose regulation. These findings suggest potential utility in diabetic wound management, although direct wound-healing effects were not established in the reported study.	[125]
**16**	Cefazolin/ZnO nanofiber mats	Nanofibrous mats; sustained drug release; MIC 1.9 ± 0.2 μg/mL for 1:1 formulation	Cefazolin + ZnO NPs	Wistar rat wound model; *S. aureus*	In vitro cytocompatibility reported	ZnO-loaded cefazolin nanofiber mats showed enhanced antibacterial activity, with the 1:1 formulation exhibiting a MIC of 1.9 ± 0.2 μg/mL against *S. aureus*, along with sustained drug release and bacterial cell-wall disruption. In vivo, the mats enhanced wound healing in Wistar rats, with histology showing improved cell adhesion, epithelial migration, and collagen synthesis.	[126]
**17**	ZnO–CuO nanocomposite hydrogel	ZnO–CuO NPs ~18.67 nm	ZnO + CuO	In vitro wound-healing model	Cytocompatibility reported	The research group developed a ZnO–CuO nanocomposite-embedded polyethylene glycol hydrogel (CPZCH) that had superior characteristics for wound care applications, including high swelling and porosity, controlled degradation, strong antibacterial activity against common pathogens within 24 h, high cytocompatibility, and enhanced fibroblast-mediated wound closure in vitro, suggesting that CPZCH has great potential as an effective, multifunctional wound dressing	[127]
**18**	SA@Cu-MEL hydrogel	Injectable/self-healing Cu-containing hydrogel	Copper ions + melittin	Infected wound model	Good mechanical properties and reported biocompatibility	SA@Cu-MEL hydrogel showed strong antibacterial activity against *S. aureus* and *E. coli*, along with antioxidant and anti-inflammatory effects through M2 macrophage polarization. The hydrogel promoted cell proliferation, migration, angiogenesis, and collagen deposition, resulting in accelerated healing of infected wounds.	[128]
**19**	Cu/F-MOF PVA/gelatin nanofibers	192 ± 8 nm composite nanofibers	HKUST-1/F-HKUST-1 Cu-MOFs	Antimicrobial + wound-healing model	Biocompatible polymer/MOF system; detailed long-term toxicity not established	PVA/gelatin nanofiber mats containing HKUST-1 and F-HKUST-1 MOFs showed strong antibacterial activity against *S. aureus*, *K. pneumoniae*, and *C. parapsilosis*, with good mechanical strength and water uptake. The biocompatible polymer–MOF combination promoted epithelialization and granulation tissue formation, supporting improved wound healing potential.	[129]

### 4.2. Carbon-Based Nanomaterials

Carbon-based nanomaterials such as graphene, graphene oxide (GO), reduced graphene oxide (rGO), carbon nanotubes (CNTs), carbon dots (CDs), fullerenes, and nanodiamonds [130] are promising candidates in wound healing because of their structural and functional characteristics, strong antimicrobial effects (due to membrane disruption and inhibition of biofilm formation), antioxidant behavior (to balance the oxidative stress at the wound site), modulation of key signaling pathways (such as VEGF for angiogenesis, TGF-β/Smad for collagen production and extracellular matrix remodeling, and PI3K/Akt and MAPK pathways for cell proliferation, migration and survival), promotion of fibroblast activation, keratinocyte migration, and re-epithelialization, and their ability to serve as a carrier for drugs or growth factors for further tissue regeneration and acceleration of wound healing [131].

The biological effects of carbon-based nanomaterials are strongly influenced by their surface chemistry, degree of oxidation, defect density, morphology, electrical conductivity, specific surface area, and capacity for chemical functionalization. These characteristics regulate adsorption of proteins and biomolecules, cellular adhesion and internalization, ROS generation or scavenging, and interactions with intracellular organelles, thereby establishing the molecular basis for their regenerative effects. Graphene-based materials and carbon nanotubes, for example, can modify cell–material interactions and intracellular redox status, which may influence MAPK and PI3K/Akt signaling involved in cell survival, proliferation, migration, and cytoskeletal organization. Their effects on ROS-sensitive inflammatory signaling can additionally influence NF-κB activity and the transition from persistent inflammation toward tissue repair. In parallel, modulation of VEGF-associated signaling can promote endothelial cell proliferation and migration and thereby support neovascularization, whereas interaction with TGF-β/Smad signaling may influence fibroblast activation and extracellular matrix deposition [132]. Surface functionalization is particularly important because pristine and functionalized carbon materials can produce substantially different biological responses; incorporation of hydrophilic polymers, biomolecules, or therapeutic cargos can alter cellular uptake, dispersibility, ROS behavior, and tissue compatibility. Consequently, the regenerative potential of carbon-based nanomaterials is determined by the balance between their structural and electronic properties and their biological interactions, with appropriate control of surface chemistry and dose being essential to obtain beneficial modulation of oxidative stress, inflammation, angiogenesis, and tissue remodeling without inducing persistent oxidative or inflammatory injury [133,134].

#### 4.2.1. Graphene and Graphene Oxide

Graphene and graphene oxide (GO)-based nanomaterials have attracted a lot of interest with regard to wound healing [135] because of their antimicrobial, anti-inflammatory, and regenerative properties, accelerating healing by interacting with key cellular signaling pathways that control tissue repair, enhancing angiogenesis through activation of the VEGF pathway, influencing the TGF-β/Smad pathway, and modulating the PI3K/Akt and MAPK signaling pathways, which are important for keratinocyte migration, cell proliferation, and survival [136]. Hussein et al. demonstrated that 1% ultrasonicated GO enhanced cell migration in an in vitro skin-scratch assay and promoted wound closure in a rat excisional skin-defect model, while supporting cell attachment and proliferation [137]. Rehman et al. further demonstrated that 0.002% (*w*/*w*) rGO incorporated into GelMA hydrogel significantly enhanced fibroblast, endothelial cell, and keratinocyte proliferation and migration and promoted angiogenesis in a chick embryo model, supporting its potential for wound regeneration [138]. Mukherjee et al. showed that GO and reduced GO possessed angiogenic activity in multiple in vitro and in vivo assays, with intracellular ROS/RNS generation and activation of phospho-eNOS and phospho-Akt identified as plausible mechanisms underlying GO-associated angiogenesis [139]. Graphene and GO materials can also regulate ROS levels and oxidative stress, while their large surface area and functional groups enable incorporation into hydrogels and other wound-dressing systems, making them promising nanoplatforms for multifunctional wound repair [136]. Elhami et al. developed chitosan/rGO nanocomposites containing curcumin, papain, and collagen peptides, showing enhanced anti-inflammatory, antioxidant, antibacterial, and cell-viability effects. In vivo, the optimized formulation improved wound healing, supporting enhanced wound repair; however, this does not establish definitive tissue regeneration or scar-free healing, and the reported superiority remains specific to the tested formulation and experimental model [140]. In another study, Hassen et al. demonstrated TRGO/ZnO nanocomposites with ROS-mediated killing of *S. aureus*, *P. aeruginosa*, and *E. coli* with good in-vitro biocompatibility. The results are applicable to the potential management of infection in wounds, but have not been shown to have direct tissue-regenerating or wound-healing effects in the reported study [141]. In another work, the multifunctional wound-dressing potential of FC-rGO-PDA was demonstrated by its good adhesion, cytocompatibility, hemostatic activity, and antibacterial properties against *S. aureus* and *E. coli*. The properties listed above do not, however, prove superior tissue regeneration or scar-free healing; more in vivo and long-term research is required to validate the regenerative effectiveness [142].

Hao et al. developed GelAlg@rGO-pEV hydrogel for diabetic wounds, with the ability to modulate macrophages, reduce ROS levels, stimulate cell migration, regulate inflammation, and promote angiogenesis. In vivo, NIR activation also lowered inflammatory biomarkers while increasing angiogenesis and heat-shock protein expression, improving the healing process, albeit not necessarily leading to scarless regeneration [143]. Injectable conductive GO hydrogel not only suppressed the local immune response by inducing the shift of M1 polarization in macrophages to an M2-like phenotype but also exhibited antibacterial properties and neovascularization. These effects in vivo promoted wound healing in infected diabetic wounds, but not complete regeneration without scarring [144]. Khan and co-workers developed bacterial cellulose/gelatin/GO hydrogels with favorable swelling and mechanical properties, controlled curcumin release, good fibroblast viability and proliferation, antibacterial activity, and excellent hemocompatibility. Their findings corroborate the gels’ potential as multifunctional wound dressings; however, the demonstrated benefits of the material remain largely in vitro, and the results should not be interpreted as definitive tissue regeneration or scar-free healing. More in vivo experiments and longer follow-up for evaluation of the regenerative effect and scar-related side effects are needed. This research work indicated that graphene and graphene oxide-based nanomaterials are a multifunctional and synergistic platform for advanced wound healing applications, with their therapeutic efficacy stemming from their ability to simultaneously modulate key cellular signaling pathways (e.g., VEGF, TGF-β/Smad, PI3K/Akt, and MAPK), regulate oxidative stress through controlled reactive oxygen species (ROS) balance, and exert intrinsic antimicrobial activity. As nanocomposites with biopolymers and bioactive agents, graphene and graphene oxide-based nanomaterials exhibit improved physicochemical properties, biocompatibility, and targeted biological responses (e.g., enhanced cell proliferation, angiogenesis, and immune modulation), enabling accelerated tissue regeneration and effective infection control, suggesting they are strong candidates for next-generation, bioactive wound dressing systems, but further long-term safety and clinical translation studies are needed.

#### 4.2.2. Carbon Nanotubes

Carbon nanotubes (CNTs) are promising nanomaterials for wound healing because of their physicochemical properties such as high surface area, mechanical strength, electrical conductivity, and ease of functionalization [145], which enable them to serve as bioactive scaffolds in wound environments for cell adhesion, proliferation, and migration, and provide antimicrobial effects through membrane disruption and oxidative stress induction in pathogens, and electrical signaling to aid tissue repair such as re-epithelialization and angiogenesis [146]. They are combined with hydrogels, nanofibers, or composite dressings to enhance structural integrity, provide controlled drug delivery, and create a moist microenvironment for healing. CNTs regulate multiple signaling pathways that are critical for wound healing at the molecular level, including the PI3K/Akt pathway that mediates cell survival, proliferation, and migration of keratinocytes and fibroblasts, the MAPK signaling cascade (ERK, JNK, and p38 pathways) that controls cell differentiation and inflammatory responses, upregulation of the VEGF signaling pathway for angiogenesis, modulation of the TGF-β/Smad pathway that is responsible for extracellular matrix deposition and tissue remodeling, and interaction with ROS to maintain redox homeostasis to minimize excessive oxidative stress while preserving signaling functions [147]. CNTs can also interact with cellular signaling involved in tissue repair. Zhang et al. reported that multi-walled CNTs (20–30 and 30–50 nm) affected endothelial cell migration and tube formation through the VEGF–Akt–eNOS axis; however, CNT exposure reduced VEGF, Akt, and eNOS expression and suppressed angiogenesis, indicating that this pathway is highly dependent on CNT concentration and biological context [148]. Therefore, CNT-based systems should be considered promising scaffold and delivery platforms, while their effects on angiogenesis, ROS, and specific signaling pathways require careful consideration of nanotube type, functionalization, dose, and exposure conditions. Tan and colleagues designed and synthesized carbonized cellulose aerogels for reinforcing with CNTs, which showed high porosity, proper hydrophilicity, shape recovery, cytocompatibility, and hemocompatibility. The aerogels were shown to promote platelet activation and clot formation, control bleeding in rat models, and facilitate wound healing similar to the commercial dressing; beneficial effects in terms of tissue regeneration and scar-free healing were shown [149]. In another study, a novel multifunctional microneedle patch was developed based on aligned CNT sheets and HA to offer excellent biocompatibility, biodegradability, and safety. The CNT layer provided a highly ordered structure for photo-thermal and electro-thermal effects. It was able to release therapeutic agents in a controlled manner, guided fibroblast cells to the right orientation for tissue regeneration, and incorporated VEGF to enhance the formation of endothelial tubular structures for angiogenesis, as shown by experimental results in animal models, which all suggest that this CNT-integrated, VEGF-loaded microneedle patch is an advanced strategy for wound repair and regeneration [150]. Tavakoli et al. manufactured a trilayer wound dressing composed of PAAm–Aloe vera/MWCNT, which demonstrated enhanced cell viability, adhesion, and angiogenesis, controlled release of IGF1, and improved mechanical strength. The optimized Trilayer0.5 dressing produced faster wound healing in 10 days in vivo, which indicated better healing of wounds, but does not necessarily mean that the wound was completely re-epithelialized or that there was no scar formation [151]. Tavakoli et al. developed a GG/ZnONP–MWCNT bionanocomposite film with enhanced fluid absorption and strong antibacterial activity. In vivo, the film achieved 100% wound closure within 14 days, compared with lower healing rates for pure GG and untreated controls, indicating improved wound healing. However, this result was model- and formulation-specific and should not be generalized as superior to other nanomaterials or interpreted as definitive tissue regeneration [152]. In another work, a new multifunctional nanozyme was designed by introducing FeCo alloys into carbon spheres and carbon nanotubes (FeCo-C/CNT nanocomposites) to enhance their antibacterial activity and wound healing. The FeCo-C/CNT nanocomposites exhibited superior oxidase-like activity and photothermal properties and thus had a combined therapeutic effect with high antibacterial efficiency against Staphylococcus aureus and *Escherichia coli* in vitro, which was mainly attributed to the synergistic effect of ROS generation and heat under NIR irradiation and further promoted by ROS production. FeCo-C/CNT nanozymes were also used to heal infected wound models, showing hydrophobic protection and combining catalytic and photothermal therapy, leading to superior wound recovery. Collectively, these results demonstrate that FeCo-C/CNT nanozymes may serve as a promising strategy for effective treatment of wound healing [153].

#### 4.2.3. Carbon Dots

Carbon dots (CDs) are nanoscale carbon-based materials that have been demonstrated to be promising nanomaterials for wound healing, as they have good biocompatibility, are strong antioxidants, have intrinsic antimicrobial effects, and reduce oxidative stress by scavenging ROS to reduce tissue damage and inflammation at the wound site [154]. CDs can also promote fibroblast proliferation, collagen deposition, and angiogenesis and are associated with the modulation of critical signaling pathways, such as the PI3K/Akt pathway for cell survival and proliferation, the VEGF pathway for angiogenesis, and the TGF-β pathway for extracellular matrix remodeling and scar reduction [155]. CDs also regulate inflammatory responses through pathways like NF-κB, maintaining a balanced healing environment. In one study, Qu et al. prepared positively charged carbon dots (CDs) with their excellent biocompatibility, strong antibacterial activity against *S. aureus*, and ROS-scavenging activity. In a mouse skin infection system, wound healing was quicker after topical application of CDs, and this correlated with their antimicrobial and antioxidant properties in wound healing, but this does not constitute scar-free regeneration. The findings should be considered in the context of the specific wound model and the specific treatment conditions, and further studies are needed to assess long-term tissue remodeling and collagen organization and to verify scar outcomes [156]. In another study, cerium-doped carbon nanodots (Ce-CNDs) demonstrated excellent biocompatibility, moderate antibacterial properties, and potent antioxidant capacity due to the Ce^3+^/Ce^4+^ redox states, which helped to scavenge excess ROS and decrease oxidative stress. The Ce-CNDs demonstrated acceleration of wound healing, which was attributed to their potential in terms of antioxidant activity, but this result was not sufficient to confirm the scar-free regeneration process. More research is needed to test for tissue remodeling over time, organization of collagen, and validated scar outcomes [157]. Zhao et al. designed zinc-doped curcumin carbon dots (CCDs), which created ROS molecules for light-activated PDT and also exhibited cell proliferation, migration, angiogenesis, and collagen formation properties with antibacterial activity. VEGF signaling was found to be involved in the pro-angiogenic response and played a role in accelerated healing of infected wounds, but the results did not unequivocally demonstrate scar-free regeneration. Long-term tissue remodeling and collagen organization, as well as validated scar outcomes, should be evaluated further [158]. Ren et al., developed a multifunctional autologous platelet concentrate-based platform (CurCDs@iPRF-MA) for enhancing chronic wound healing in diabetic conditions, combining injectable platelet-rich fibrin (iPRF), GelMA, and curcumin-derived carbon dots (CurCDs), enabling easy injection followed by light-induced gel formation for better clinical applicability. Their research showed improved therapeutic performance achieved by efficiently scavenging reactive oxygen species and attenuating inflammation, regulating mitochondrial function, and activating oxidative phosphorylation for enhanced cellular metabolism, promoting macrophage polarization toward a healing-supportive phenotype and enhanced vascularization through sustained autologous growth factors release, which significantly enhanced wound healing in diabetic models. The results indicate this strategy as a promising, controllable, and multifunctional strategy for advanced wound healing [159]. Li et al. prepared Fe/N-doped chitosan-chelated carbon dot nanozymes (CS@Fe-N CDs) with high antibacterial activity as peroxidase mimics that exhibited over 2000-fold higher activity against *S. aureus* in the presence of the nanozyme, with negligible cytotoxicity. This was model-dependent, and no such superiority of the nanozyme over antibiotics was established in vivo; however, in vivo, the nanozyme accelerated wound healing in rats compared to conventional antibiotic treatment, although this did not necessarily result in scar-free regeneration [160]. Guo et al. created EUO-NAC-CDs that decreased pro-inflammatory cytokines (TNF-α, IL-6, IL-1β), increased expression of the anti-inflammatory cytokine IL-10, and promoted angiogenesis and tissue repair by altering the expression of CD31, VEGF, CD206, and iNOS. Although the CDs improved wound healing and added bioimaging capabilities, these results do not indicate completely scar-free regeneration, and long-term studies of tissue remodeling, collagen organization, and scar outcomes are needed for confirmation [161]. In another study, a multifunctional bacterial cellulose-based composite membrane loaded with copper-doped carbon dots (BC/Cu(II)-RCDs) was developed to solve problems in wound healing, including infection, inflammation, oxidative stress, and angiogenesis. Carbon dots were successfully loaded into the BC matrix to improve the hydrophilicity, mechanical strength, and thermal stability of the membrane. The in vitro tests showed good biocompatibility, strong antibacterial and anti-inflammatory activities, and enhanced angiogenesis, while in vivo studies demonstrated accelerated wound healing, as indicated by enhanced epithelial regeneration, collagen deposition, and tissue formation, and reduced inflammation in infected wounds. The composite membrane was found to activate VEGF and MAPK signaling pathways, which are involved in cell migration, blood vessel formation, and tissue regeneration [162].

### 4.3. Polymeric Nanomaterials

Polymeric nanomaterials stimulate wound healing through interactions with and modulation of several of the major cellular signaling pathways that are responsible for controlling inflammation, cell proliferation, angiogenesis, and tissue remodeling [163,164], including the PI3K/Akt signaling pathway that promotes cell survival, migration, and proliferation of fibroblasts and keratinocytes, the VEGF signaling pathway that is important for angiogenesis and new blood vessel formation, and the TGF-β/Smad pathway that is critical for extracellular matrix deposition, collagen synthesis, and scar formation [165].

Sharda et al. demonstrated that approximately 30-nm insulin-loaded chitosan nanoparticles significantly accelerated healing of third-degree burn wounds in mice, with reduced IL-6, increased IL-10, enhanced re-epithelialization, and greater collagen deposition, while modulation of the Nrf2 pathway was associated with improved antioxidant response [166]. Sun et al. further demonstrated that rebamipide-loaded chitosan nanoparticles accelerated postoperative wound repair and re-epithelialization by suppressing M1 macrophage polarization and NF-κB activation, accompanied by reduced IL-1β, IL-6, IL-12, and TNF-α levels [167]. In addition, the PLGA-VEGF nanoparticle system developed by Cao et al. significantly enhanced granulation tissue formation, collagen content, re-epithelialization, and angiogenesis in both non-diabetic and diabetic mouse wounds; the nanoparticles also enhanced keratinocyte proliferation and migration and increased VEGFR2 expression [168]. The NF-κB pathway is frequently inhibited to decrease excessive inflammation and promote a healthy immune response, while activation of the MAPK pathway (ERK, JNK, and p38) facilitates cell growth, differentiation, and stress response to facilitate healing. Some of the more advanced polymeric systems modulate the Wnt/β-catenin pathway to promote tissue regeneration and re-epithelialization. Collectively, these nanomaterials establish a conducive microenvironment for enhanced and rapid wound healing via the coordination of multiple signaling pathways [39,169].

The therapeutic performance of polymeric nanomaterials is fundamentally dependent on polymer composition, molecular weight, degradation behavior, particle size, surface charge, morphology, drug-loading capacity, and release kinetics. These parameters determine nanoparticle stability in the wound environment, cellular uptake, intracellular trafficking, tissue retention, and the temporal concentration of therapeutic molecules available to specific cellular populations. Consequently, polymeric nanomaterials provide an important platform for regulating signaling pathways through controlled and localized delivery rather than simply exposing wound tissue to a constant drug concentration [170]. Following cellular internalization or local cargo release, therapeutic agents delivered by polymeric systems can influence PI3K/Akt signaling, thereby promoting cell survival, proliferation, migration, and metabolic adaptation; MAPK/ERK, JNK, and p38 signaling, which regulate proliferation, differentiation, stress responses, and inflammatory activation; and NF-κB signaling, which is particularly important for controlling excessive inflammatory mediator production. Polymeric systems can also influence VEGF-dependent angiogenic responses and TGF-β/Smad and Wnt/β-catenin signaling involved in fibroblast behavior, extracellular matrix organization, epithelial regeneration, and tissue remodeling. Importantly, the degradability and release characteristics of the polymer determine how long these pathways remain exposed to the therapeutic stimulus. A rapidly releasing formulation may produce a strong but transient response, whereas a slowly degrading polymer may maintain pathway modulation for prolonged periods, which may be beneficial for sustained repair but potentially detrimental if profibrotic or anti-inflammatory effects persist beyond the appropriate healing phase. Therefore, optimization of polymer architecture and release kinetics is critical for achieving pathway-specific therapeutic activity while maintaining temporal compatibility with the different stages of wound healing [171].

#### 4.3.1. Chitosan-Based Nanoparticles

Chitosan-based nanoparticles are also being explored as biomaterials for wound healing because of their intrinsic biocompatibility, biodegradability, antimicrobial activity, and ability to promote tissue regeneration [172,173]. Chitosan is a natural polysaccharide with a positive charge, which allows it to interact strongly with negatively charged cell membranes and extracellular matrix components, promoting cell adhesion and proliferation [174,175]. Formulated into nanoparticles, chitosan enhances the delivery and controlled release of bioactive agents such as growth factors, antibiotics, and anti-inflammatory compounds at the wound site, leading to hemostasis, reduced infection, and re-epithelialization and collagen deposition. Mechanistically, chitosan-based nanoparticles are known to modulate several key signaling pathways involved in wound repair, including the PI3K/Akt pathway (which promotes cell survival, proliferation, and migration of fibroblasts and keratinocytes), the MAPK pathway (to enhance cellular responses such as proliferation and differentiation needed for tissue regeneration), the NF-kB pathway (which decreases pro-inflammatory cytokine production), the TGF-beta signaling pathway (which regulates collagen synthesis, extracellular matrix formation, and scar remodeling), and the VEGF signaling pathway (which promotes angiogenesis and increased blood supply to the healing tissue). These pathways suggest that chitosan-based nanoparticle systems may contribute to the faster and more effective wound healing observed in various studies. In one study, Fahimirad et al. produced PCL/CS/CUR nanofibers with improved antibacterial, antioxidant, cell-viability, swelling, and water-vapor properties that were loaded with curcumin-loaded chitosan nanoparticles. In vivo, the modified nanofibers promoted more organized tissue repair and enhanced the healing of MRSA-infected wounds, but the results should not be viewed as scar-free regeneration, with further studies required to assess the organization of collagen, tissue remodeling, and the outcome of the scar over time [176]. In this study, Shao et al. examined the release of AgNPs from the chitosan membranes and found that the biological environment is an important factor in governing the release of AgNPs, and the higher the AgNP loading, the more effective the in vivo antibacterial activity. Overall, no negative impact was seen on the rate of wound healing or tissue reaction associated with AgNP incorporation that would suggest a detrimental effect on the biological process; however, the results do not provide evidence of enhanced tissue regeneration or scar-free healing and are formulation- and biological-environment dependent [177]. Pourseif et al. prepared chitosan-coated niosomes (TCH-Nio@CS) that showed significant improvements in sustained release and enhanced antibacterial and antibiofilm activity against *E. coli*, *P. aeruginosa*, and *S. aureus*, with ~2- and ~4-fold higher activity, respectively, compared with TCH-Nio and TCH. The system was noncytotoxic on fibroblasts and had increased infection-control potential, but it was directly proven to be tissue-regenerating or to enable healing without scarring, and these features should therefore not be assumed [178]. In another study, a bilayer electrospun membrane consisting of a cellulose acetate/polyurethane (CA/PU) outer layer for mechanical strength and a polyvinyl alcohol (PVA) inner layer embedded with ciprofloxacin-loaded chitosan nanoparticles for antibacterial activity was successfully developed, which showed desirable physicochemical properties (high porosity, hydrophilicity, increased crystallinity, and good thermal and mechanical stability). The morphology was confirmed by the formation of uniform nanofibers with reduced fiber diameter upon nanoparticle incorporation, and the drug release study demonstrated sustained ciprofloxacin release from nanoparticle-loaded membranes and showed strong antibacterial activity, excellent hemocompatibility, and high cell viability, promoting cell attachment and migration. These results indicate the promising potential of the developed electrospun membranes as effective materials for wound healing and tissue engineering [179]. Liu and colleagues developed a carbon nitride-polydopamine-silver complex (C3N4-PDA-Ag@CS) composite film, which is a very effective antibacterial wound dressing and that shows dramatically enhanced antibacterial activity against common pathogens *Staphylococcus aureus* and *Pseudomonas aeruginosa*. In addition to superior antimicrobial performance, the composite also showed good biocompatibility, as evidenced by hemolysis, cytotoxicity, and in vivo implantation tests. The dressing can also speed up the healing of infected models by enhancing collagen deposition and epidermal regeneration. The results showed that this composite can serve as an alternative to antibiotic-based treatment for infection control and tissue repair [180]. Aassar et al. prepared a chitosan-based gelatin/PVP membrane with enhanced mechanical strength, hydrolytic stability, and antibacterial efficacy (1.5–3 cm inhibition zone) against pathogenic bacteria by embedding ~9.9nm AgNPs in the matrix. The membrane was also found to decrease antibiotic-resistance genes, which is also promising for infection control; however, these results do not prove improved tissue repair or scar-free healing, and additional in vivo and long-term studies are still needed to substantiate wound repair and scar outcomes [181]. Song and colleagues developed a novel chitosan-based metal–organic polyhedrons (MOPs)/enzyme hybrid hydrogel for wound healing using crosslinking glucose oxidase (GOx), vanadium-based MOPs (VMOP-2), and chitosan with glutaraldehyde as a crosslinking agent. Characterization using FTIR, SEM, XPS, TGA, and EDX confirmed the successful formation of the GVCS hydrogel, with homogeneous distribution of GOx and VMOP-2 in the hydrogel. The hydrogel showed strong antibacterial activity against both Gram-positive and Gram-negative bacteria owing to its ability to generate hydroxyl radicals in the presence of glucose, and it also exhibited good biocompatibility (MTT assay) and promoted wound healing in in vivo studies using an infected wound model. The developed GVCS hydrogel shows great promise as a novel antibacterial material for clinical wound healing [182]. In another study, Deng et al. prepared a quaternized chitosan/dialdehyde bacterial cellulose (BDC) hydrogel with rapid self-healing, injectability properties, and excellent water-retaining and biocompatible properties, which also has good antibacterial activity against *E. coli* and *S. aureus*. The hydrogel was found to stimulate cell growth and spreading, which are beneficial for wound healing and tissue repair. In general, the combination of antibacterial efficacy, ECM-mimicking, and self-healing abilities makes it a candidate for wound dressing, but its healing ability should be considered in the context of the specific formulation and experimental model [183]. Deng et al. demonstrated that quercetin-loaded PLGA nanoparticles and pluronic thermosensitive gel accelerated the healing process of burn wounds, whereas the quercetin solution and Silverdin^®^ had no effect. The thermosensitive gel demonstrated superior drug release and overall therapeutic activity, with beneficial effects on growth factors, cytokines, granulation tissue, and inflammation management, while the PLGA nanoparticles exhibited low drug encapsulation efficiency (25 ± 5%) and limited drug release. The results indicated that the thermosensitive gel was a more effective and effective formulation for burn wound healing under the tested conditions [184].

#### 4.3.2. PLGA Nanocarriers

PLGA-based nanocarriers have been widely studied for wound healing applications because of their biodegradability, biocompatibility, and ability to provide controlled drug delivery [185]; PLGA is an FDA-approved biodegradable polymer that degrades into non-toxic byproducts and can deliver growth factors, antibiotics, and anti-inflammatory agents to the wound site to facilitate healing by accelerating the inflammatory and proliferative phases through stimulation of fibroblast activity, collagen deposition, and tissue regeneration [186]. PLGA systems can affect important pathways at the molecular level, including the Wnt/β-catenin signaling pathway, which controls cell proliferation and migration, the TGF-β signaling pathway, which controls extracellular matrix formation and remodeling, the VEGF signaling pathway to enhance angiogenesis for proper blood supply to healing tissue, modulation of the NF-κB signaling pathway to decrease excess inflammation, the PI3K/Akt signaling pathway, and the MAPK signaling pathway to support cell survival and proliferation, and the HIF-1α signaling pathway to promote angiogenesis and tissue repair under hypoxic conditions [187]. Thus, PLGA nanocarriers represent a promising therapeutic approach for advanced wound healing applications due to their ability to provide targeted, sustained, and efficient therapies [185]. In one study, PLGA and chitosan-coated PLGA nanoparticles loaded with vitamin A palmitate (VAP) were developed using the nanoprecipitation method, with desirable physicochemical properties, a size range of approximately 196–669 nm, and low polydispersity index values, indicating high-quality and uniform formulations. Surface charge analysis showed that uncoated PLGA nanoparticles had a negative ζ-potential, while chitosan-coated nanoparticles had a positive ζ-potential, confirming successful coating. In vitro release studies indicated an initial burst release followed by a sustained release pattern, which may be beneficial for sustained therapeutic action. Among all the tested formulations, NP-7 showed the most effective performance, probably owing to its smaller particle size and chitosan coating, which enhanced drug delivery efficiency. Biological evaluation using HaCaT keratinocyte cells showed that NP-7 significantly enhanced cell proliferation and wound healing [188]. In another work, Nasrullah developed CAPE-loaded nanoparticles (~198 nm) that significantly accelerated wound closure in diabetic rats compared with free CAPE. The formulation was shown to improve the deposition of collagen and hydroxyproline content, reduce lipid peroxidation, preserve SOD and CAT, decrease the expression of IL-6 and TNF-α, and increase the expression of Col1A1, thereby promoting wound healing through these activities [189]. Dhal et al. prepared the gentamicin sulfate-loaded PLGA nanoparticle pullulan film (PNP-F), which showed sustained release of gentamicin (86.76% over 192 h) and had effective antibacterial activities against *P. aeruginosa* and *S. aureus*. The film facilitated dermal fibroblast migration and proliferation in vitro and significantly better healing of wounds in an in vivo setting compared with marketed gentamicin cream, suggesting that the film could also be beneficial in wound repair and infection control [190]. In another study, Wang et al. fabricated a core–shell PLGA nanofibrous scaffold, with ZnO NPs and VEGF as the core and shell, respectively, which exhibited excellent antibacterial ability, cytocompatibility, and sustained dual-agent delivery. The results of the combined system showed that it improved wound healing by facilitating accelerated wound contraction, better tissue organization, and more neovascularization, indicating its potential application as a multifunctional dressing for infected wounds in vitro and in vivo [191]. In another study, authors showed that harmala alkaloid-rich fraction (HARF) encapsulated in chitosan-coated PLGA nanoparticles (H/CS/PLGA NPs) represents a potent and synergistic system for the treatment of infected wounds. The optimized nanoparticles displayed favorable physicochemical properties, such as nanoscale size, good uniformity, positive surface charge, and high entrapment efficiency, and a biphasic drug release profile with an initial burst and sustained release. The formulation was found to be functionally more active, with significantly increased antibacterial activity against *Staphylococcus aureus* and *Escherichia coli* than free HARF, indicating enhanced therapeutic potency, and it was also biocompatible with human skin fibroblasts, suggesting its safety. In vitro wound healing studies showed a markedly higher wound closure rate with the nanoformulation compared to free drug and blank nanoparticles. The combination of PLGA, chitosan, and HARF produced an effective and synergistic system for managing infected wounds [192]. In another study, Basaran et al. prepared sericin/gelatin nanofibers loaded with heparin nanoparticles and accomplished 30.04 mg/g loading capacity and 60% encapsulation efficiency, whereby the release of heparin was controlled. The 1:2 ratio of sericin/gelatin scaffold exhibited a lower degradation rate, high water retention, and favorable fiber morphology, conducive to controlled delivery and skin tissue regeneration [193]. Zhang et al. developed a PLGA@IL-8/ADM scaffold that enhanced MSC proliferation, survival, and endothelial differentiation in the diabetic wound environment. The system facilitated capillary formation, collagen deposition, tissue repair, and upregulation of VEGF and α-SMA expression in vivo, which were associated with enhanced diabetic wound healing, but this was observed only in the tested MSC-based scaffold via a mouse model [194]. Gourishetti and colleagues developed a PLGA@IL-8/ADM scaffold to support MSC-based therapy in diabetic wounds. In vivo, the system was found to boost the proliferation, survival, and endothelial differentiation of MSCs; it increased the formation of capillaries, collagen deposition, tissue regeneration, and expression of VEGF/α-SMA, contributing to better diabetic wound healing; but these effects were limited to the tested scaffold in a mouse model [195].

#### 4.3.3. Silk Fibroin Nanomaterial

Silk fibroin nanomaterials have emerged as highly promising biomaterials for advanced wound healing due to their excellent biocompatibility, biodegradability, mechanical strength, and ability to support cell adhesion and proliferation [196]. In nano-formulations such as nanofibers, nanoparticles, and hydrogels, silk fibroin provides a moist healing environment, promotes re-epithelialization, and can be engineered for controlled delivery of drugs, growth factors, or antimicrobial agents [197]. These systems actively modulate key signaling pathways involved in wound repair, including the PI3K/Akt pathway, which enhances cell survival and proliferation; the MAPK/ERK pathway, which regulates cell migration and differentiation; the TGF-β/Smad pathway, crucial for collagen synthesis and tissue remodeling; and the NF-κB pathway, which controls inflammation [39]. In addition, silk fibroin-based materials can also stimulate angiogenesis by upregulating VEGF signaling. Thus, silk fibroin nanomaterials regulate multiple interconnected pathways that not only accelerate wound closure but also enhance the quality of regenerated tissue, making them an attractive platform for next-generation wound healing therapies [198]. In one study, Arumugam and colleagues fabricated an advanced wound healing material, silk fibroin/cellulose acetate (SF/CA) composite nanofibers with palladium (Pd) and platinum (Pt) nanoparticles. The nanofibrous scaffold had a well-interconnected structure with nanoscale fiber diameter and uniformly distributed Pd and Pt nanoparticles, which provided desirable physical and mechanical properties such as high porosity, swelling capacity, and appropriate degradation for exudate absorption, cell adhesion, and tissue regeneration. The in vitro results indicated excellent antibacterial activity against *Escherichia coli* and *Staphylococcus aureus*, good biocompatibility, and cell migration and proliferation in fibroblast cells. In vivo studies in rats indicated wound healing efficiency with more than 99% closure, with high hemocompatibility, supported by histological analysis showing enhanced re-epithelialization, granulation tissue formation, angiogenesis, and reduced inflammation [199].

Heidari and colleagues developed an SF/PVA nanofiber dressing incorporating RGO and ZnO nanoparticles, showing strong antibacterial activity, good fibroblast viability, proliferation, and migration, along with improved mechanical and surface properties. These nanofibers proved to be a multifunctional wound dressing because of their ability to promote wound healing and tissue repair in vivo, but the proven effects were limited to the tested formulation and animal model [200]. In another study, a silk fibroin-based scaffold incorporating self-assembled nanoparticles was developed to improve hemostasis and cell adhesion during wound healing. The nanoparticles were evenly distributed in the scaffold, which increased the surface roughness and structural properties, including decreased pore size, increased compressive strength, slower degradation, and maintained water absorption capacity. The modified scaffold exhibited better hemostatic performance (with a higher blood clotting index and better platelet adhesion) compared with the pure scaffold. In vitro results also confirmed good biocompatibility and increased cell proliferation, migration, and adhesion of L929 fibroblasts and HUVECs. The prepared nanoparticle-loaded scaffold has strong potential as an effective wound dressing promoting rapid hemostasis and subsequent tissue regeneration [201]. Yu et al. prepared a nanocomposite hydrogel composed of thiolated hyaluronic acid, silk fibroin, and bioactive glass that exhibited superior injectability, mechanical strength, and controlled Si-ion release for ~3 weeks. In vivo, the hydrogel facilitated rapid and complete wound healing in 2 weeks, and in vitro, promoted the migration and proliferation of fibroblasts and endothelial cells, favoring wound healing and tissue regeneration [202]. In another work, a 3D-printable multifunctional hydrogel (STFWA) was developed to treat bacterial wound infections more effectively. The hydrogel has a dual-network structure (silk fibroin and tannic acid–Fe^3+^) with W_18_O_49−x_@Au nanostructures that form a Schottky heterojunction, enhancing charge separation and ROS generation, and shows enhanced photothermal performance due to dual LSPR and strong light absorption. The hydrogel exhibits effective antibacterial activity against Staphylococcus aureus and Escherichia coli under near-infrared irradiation via combined photothermal and photodynamic effects. In vivo studies showed faster wound healing, better angiogenesis, and controlled inflammation, which suggests potential for clinical wound care applications [203]. Cheng et al. reported a stable and biocompatible liquid metal-based hydrogel (RSF-P-EGaIn) for antibacterial wound healing. To address the instability of liquid metal, an environmentally friendly ice-bath ultrasonic approach was developed to prepare regenerated silk fibroin-coated EGaIn nanoparticles (RSF@EGaIn NPs), which exhibited good structural stability. The RSF@EGaIn NPs were embedded into a double-crosslinked hydrogel with PNIPAAm, providing mechanical strength and temperature responsiveness. The hydrogel exhibited greatly enhanced antibacterial performance under near-infrared irradiation, and in vivo studies showed complete wound healing within 14 days with new skin and hair formation, enhanced epithelialization, and orderly deposited collagen. This system holds great promise for photothermal-responsive wound healing and potential future drug delivery [204].

### 4.4. Lipid-Based Nanocarriers

Lipid-based nanocarriers such as liposomes, solid lipid nanoparticles, and nanostructured lipid carriers are being increasingly studied for their use in wound healing [205] as they improve drug delivery, stability, and therapeutic efficacy by encapsulating a variety of bioactive agents including antibiotics, growth factors, and anti-inflammatory compounds for controlled and prolonged release at the wound site, thereby modulating key signaling pathways that drive cell proliferation and migration (e.g., the PI3K/Akt signaling pathway and MAPK/ERK pathway), reducing excessive inflammation (e.g., the NF-κB signaling pathway), promoting angiogenesis (e.g., the VEGF signaling pathway), and regulating extracellular matrix remodeling (e.g., the TGF-β signaling pathway) to facilitate enhanced tissue regeneration. Lipid-based nanocarriers have distinct physicochemical and biological properties that directly contribute to wound healing. They are biocompatible and biodegradable, reducing toxicity and making them appropriate for the sensitive wound environment; the lipid composition closely resembles biological membranes, facilitating cellular uptake and interaction with skin tissues [205]; they retain moisture well, maintaining a moist wound environment necessary for rapid healing and enhancing antimicrobial efficacy; and they can be engineered to respond to stimuli (e.g., pH-, temperature-, or enzyme-sensitive release), delivering drugs as needed [206,207]. Lipid-based nanocarriers may improve drug deposition and interaction with wound-associated tissues and biofilms; however, their penetration and distribution are formulation- and model-dependent and are influenced by particle size, surface characteristics, lipid composition, drug loading, and the biological environment. Therefore, claims of enhanced deep-tissue or biofilm penetration should be interpreted cautiously and supported by appropriate experimental evidence. High biocompatibility and biodegradability, along with the protection of encapsulated drugs, controlled release, and enhanced permeation, make lipid-based nanocarriers ideal multifunctional systems. Değim et al. demonstrated that EGF-loaded multilamellar liposomes incorporated into chitosan gel enhanced healing of second-degree burn wounds in rats, with histological and immunohistochemical analyses showing improved tissue regeneration compared with control formulations [208]. Similarly, Natarajan et al. reported that a pioglitazone-loaded nanostructured lipid carrier incorporated into a collagen/chitosan scaffold produced sustained drug release, enhanced cell growth, and significantly increased wound contraction (*p* < 0.001) in streptozotocin-induced diabetic rats, while significantly reducing MMP-9 levels (*p* < 0.001) [208].

Lipid-based nanocarriers, including liposomes, solid lipid nanoparticles, and nanostructured lipid carriers, provide a distinct mechanism of therapeutic regulation because their lipid composition, membrane-like architecture, particle size, surface characteristics, encapsulation efficiency, and lipid degradation behavior determine drug protection, cellular interaction, tissue retention, and release kinetics. Their structural similarity to biological membranes can facilitate interaction with skin cells and may improve the delivery of hydrophobic or poorly soluble therapeutic molecules to the wound microenvironment [205]. Once localized within the wound, controlled release of the encapsulated cargo can modulate PI3K/Akt and MAPK/ERK signaling involved in cell survival, proliferation, migration, and tissue regeneration, while regulation of NF-κB activity can reduce excessive inflammatory signaling. Lipid-based systems can additionally influence VEGF-associated angiogenic signaling and TGF-β-related extracellular matrix responses, thereby linking controlled drug delivery with vascularization and tissue remodeling. The temporal characteristics of lipid degradation and cargo release are particularly relevant because continuous exposure to pathway-modulating agents may not be desirable throughout all phases of healing. For this reason, sustained, localized, or stimulus-responsive lipid systems may provide greater therapeutic precision by maintaining effective concentrations at the wound site while minimizing unnecessary systemic exposure and off-target pathway modulation [209]. The overall regenerative outcome therefore depends on the interaction between lipid-carrier characteristics, cargo properties, release kinetics, and the biological stage of wound healing.

In one study, Arantes and co-workers developed ATRA-loaded solid lipid nanoparticles (~**83 nm**, ~**98% entrapment efficiency**) incorporated into chitosan films, providing controlled drug release and improved ATRA stability. The formulation was shown to significantly promote diabetic wound healing in vivo by accelerating diabetic wound closure, decreasing inflammation, increasing collagen deposition, and decreasing scar formation; however, these effects were limited to the formulation tested and the diabetic mouse model [210]. In another study, Makhmalzadeh and colleagues developed SOD-loaded SLNs with a particle size of 35–85 nm, ~78% entrapment efficiency, and ~90% retained enzymatic activity. In vivo, the formulation had a higher wound contraction rate and accelerated wound healing compared to free SOD and blank SLNs, indicating improved angiogenesis, granulation tissue, and re-epithelialization, and decreased infiltration of inflammatory cells, resulting in the promotion of burn wound healing [211]. El-Salamouni et al. formulated valsartan-loaded SLNs for topical delivery for the treatment of diabetic foot ulcers, demonstrating prolonged release and significant antibiofilm activity similar to levofloxacin. The Val-SLN formulation showed improved wound healing, decreased inflammation and microbial load in vivo, promoted re-epithelialization and collagen deposition, and regulated COX-2, NF-κB, NO, TGF-β, MMPs, and VEGF, which could help improve diabetic wound healing [212]. Farmoudeh et al. formulated SLNs containing methylene blue (SLN-MB) for controlled and sustained drug release, with an average size of 183.5 nm and 75% entrapment efficiency. These advantages indicate that MB-SLNs might have great potential for improved burn wound healing in vivo, as they facilitated re-epithelialization, tissue regeneration, and restoration of the skin layers, while also accelerating burn wound closure compared to the conventional MB gel and untreated controls [213]. Ghodrati et al. formulated NLCs loaded with PEO, which exhibited a narrow polydispersity index (PDI) and nanoscale particle size (40–250 nm), and were found to be antibacterial against both *S. aureus* and *P. aeruginosa* strains. In vivo, PEO-NLCs promoted wound contraction, decreased bacterial counts, and increased infiltration of fibroblasts, collagen deposition, re-epithelialization, and upregulation of FGF-2 and EGF, which suggested better healing of infected wounds [214]. Elkhateeb et al. showed that encapsulation of curcumin from *Curcuma longa* in nanostructured lipid carriers (CURC-NLCs) significantly increased its therapeutic potential in wound healing applications. CURC-NLCs had increased phenolic and flavonoid content and showed superior antioxidant activity compared to free curcumin. The nanoformulation also exhibited a broad-spectrum antimicrobial effect with approximately two-fold greater efficacy than native curcumin against Gram-positive, Gram-negative, and fungal strains. In vivo studies using a rabbit full-thickness wound model showed that CURC-NLCs significantly increased wound closure, with 1.15- and 1.9-fold greater healing rates compared to curcumin and untreated controls, respectively (*p* < 0.0001). Overall, encapsulating curcumin in lipid-based nanostructures significantly enhances its bioavailability and biological activity [215]. In another study, de Barros et al. prepared quercetin-loaded plant-oil-based NLCs with a nanometer size range (less than 200 nm), high zeta potential (around −40 mV), and 99% encapsulation efficiency. The QR-NLCs exhibited lower cytotoxicity and higher antioxidant and antimicrobial activities than free quercetin, especially against *S. aureus*, and improved cellular uptake and intranuclear localization, which can facilitate better topical delivery potential [216].

## 5. Smart and Stimuli-Responsive Nano-Enabled Wound Dressings

**Figure 3:**
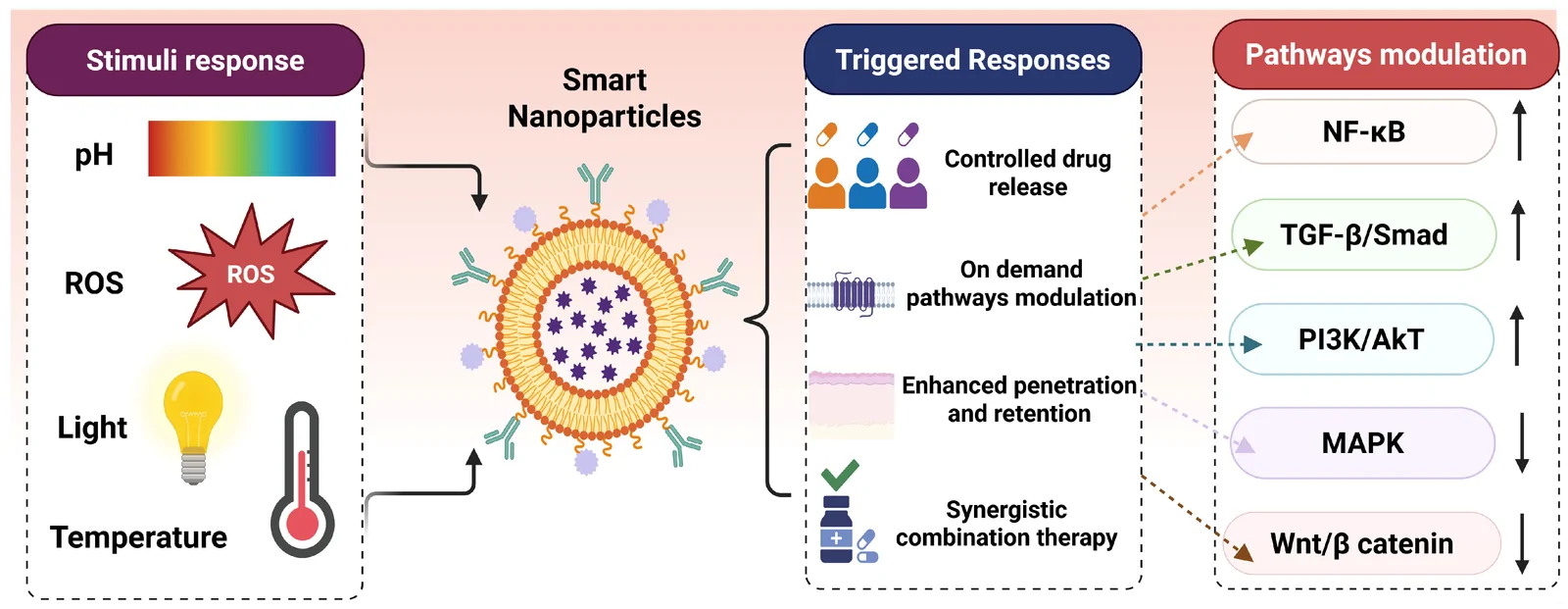
Stimuli-responsive nanomaterials for controlled modulation of wound-healing pathways. Wound-associated or external stimuli, including pH, ROS, light, and temperature, can trigger localized therapeutic responses and modulation of signaling pathways involved in inflammation, proliferation, and tissue remodeling. Pathway responses are formulation-, dose-, and wound-stage-dependent.

Smart and stimuli-responsive nano-enabled wound dressings are designed to dynamically interact with the wound microenvironment and deliver therapeutics in a controlled, on-demand manner, thereby addressing the limitations of conventional passive dressings [217,218]. These systems incorporate nanomaterials, including nanoparticles, nanofibers, hydrogels, or nanostructured lipid carriers that sense specific physiological cues related to delayed healing, such as pH shifts, increased reactive oxygen species (ROS), enzymatic activity, temperature changes, or bacterial toxins. When a specific stimulus (often present in chronic or infected wounds), such as a pH shift, increased ROS, enzymatic activity, temperature, or bacterial toxins, is detected, the dressing undergoes a structural or chemical modification that elicits localized release of entrapped bioactive agents such as antimicrobials, antioxidants, growth factors, or anti-inflammatory compounds [219]. Figure 3 is a diagrammatic representation of stimuli-responsive nanomaterials that trigger wound healing by modulating targeted pathways. This targeted and responsive release assists in controlling infection, alleviating oxidative stress, regulating inflammation, and promoting regeneration (angiogenesis, collagen deposition, and re-epithelialization), while some more advanced systems also include real-time sensing and feedback mechanisms that can monitor the wound status continuously and adjust the treatment as needed, resulting in more effective healing, reduced systemic side effects, and the establishment of individualized wound care strategies [220]. In one study, He et al. demonstrated a multifunctional, stimuli-responsive hydrogel dressing that addresses multiple pathological barriers in bacteria-infected diabetic wounds via an integrated and intelligent therapeutic strategy with dual responsiveness to near-infrared (NIR) irradiation and glucose levels for spatiotemporally controlled therapeutic action at different stages of wound healing. The incorporation of reduced graphene oxide/N, N’-di-sec-butyl-N, N’-dinitroso-1,4-phenylenediamine (rGB) and manganese dioxide (EMn) provides strong photothermal antibacterial effects for rapid infection removal by NIR exposure, while NIR-triggered nitric oxide (NO) release from rGB enhances angiogenesis, and the nanozyme activity of EMn (with catalase- and superoxide dismutase-like functions) scavenges excessive ROS and produces O_2_ to alleviate hypoxia and oxidative stress in the wound microenvironment. Additionally, the incorporation of phenylboronic acid moieties leads to glucose-responsive release of doxycycline, allowing controlled and infection-specific delivery of the antibiotic. In vivo assessment in a diabetic infected wound model indicated that this hydrogel significantly alleviated inflammation, promoted wound contraction, increased collagen deposition and neovascularization, and facilitated tissue regeneration, collectively suggesting its synergistic, multi-modal therapeutic efficacy and its potential as a next-generation smart dressing for the management of complex diabetic wounds [221].

Ghosh and co-workers developed a thermoresponsive nanofibrous dressing containing tetracycline hydrochloride and europium hydroxide nanorods that act as an antibacterial, antioxidant, and pro-angiogenic agent. In vivo, the scaffold was found to close the wound by ~95% after 21 days, while promoting increased expression of collagen-I, α-SMA, and VEGF, and decreased expression of TNF-α and IL-6, demonstrating an improved diabetic wound healing effect, specific to the tested diabetic wound model and formulation [222]. Yu et al. designed a smart wound bandage system that contained carbon dots and Fe^3^+-carbenicillin MOFs for infection sensing and responsive antibacterial therapy. The system exhibited > 99.99% antibacterial efficacy, and in vivo results demonstrated a decrease in bacterial load and a faster wound healing rate with real-time infection monitoring, while also reducing the release of antibiotics when the infection ended, which may promote more controlled management of wounds [223]. In another study, a next-generation bioadhesive hydrogel was developed to overcome the limitations of conventional wound dressings, such as poor mechanical adaptability and lack of real-time monitoring. The PVA–dextran–borax-based hydrogel with bromothymol blue (BTB) and FITC showed good and consistent tissue adhesion (~8.3 kPa), superior to commercially available dressings and suitable for dynamic, high-movement skin environments. In addition to mechanical robustness, the addition of tungsten disulfide–catechol nanozyme (CL/WS_2_) provides additional functional benefits, which may be due to catalytic and bioactive performance. This dual colorimetric sensing capability allows visual detection of infection-induced pH changes, which can be captured by a smartphone and converted into quantitative wound status data for remote, real-time monitoring without the need for sophisticated equipment, providing a practical pathway toward intelligent, patient-friendly wound management. The prepared hydrogel shows promise as a multifunctional platform that integrates adhesion, sensing, and responsiveness to enhance clinical outcomes in wound care [224].

In another work, an advanced multifunctional lignocellulose-based wound dressing was developed, integrating efficient wound healing with self-powered, intelligent sensing functions to fill a major gap in next-generation biomaterials. By utilizing ammonia–oxygen pretreatment and combining aminated lignin with tea polyphenols, the dressing was able to enhance polarity and achieve significantly enhanced triboelectric performance, enabling the system to operate as a high-output triboelectric nanogenerator for self-powered sensing applications capable of detecting multiple physiological and environmental stimuli (e.g., pressure, humidity, and material interactions) with high sensitivity, further assisted by machine learning for accurate signal interpretation. The dressing has a highly porous, interlaced fiber network with excellent air permeability and mechanical strength providing a tissue regeneration-friendly environment, and also contains polyphenols with strong antibacterial and antioxidant properties to aid in the control of infection and oxidative stress at the wound site. In vivo data show that healing is promoted (closure in 12 days) and that the status of healing can be monitored in real time without the need for external power sources. Taken together, the study presents an effective strategy for designing smart, self-powered wound dressings with both therapeutic efficacy and digital health-monitoring capabilities that can be used for future point-of-care applications [225]. The self-powered piezoelectric wound dressing showed stimulus-responsive antibiotic release with ~88.6% cumulative release under mechanical stimulation and 0% release without stimulation. The drug release and electrical stimulation achieved a healing effect 1.26 times greater than electrical stimulation alone, thereby providing precise wound management, but the findings are limited to the system tested and the experimental conditions [226]. In another study, a dual stimuli-responsive microgel-embedded hydrogel was developed to combine mechanical robustness, tissue adhesion, and controlled drug delivery for advanced wound-healing applications. Incorporation of PNIPAAm-co-AAc microgels in a polyacrylamide/chitosan semi-interpenetrating network enables the hydrogel to demonstrate stretchability, elasticity, and compressibility, allowing it to tolerate physiological stresses while maintaining close contact with different tissues. The system responds to pH and temperature changes to provide a sustained and controlled release of therapeutic agents, including a model protein (BSA) and the antibiotic sulfamethoxazole, over a long period of time. The ability to deliver two drugs with high drug-loading efficiency (>80%) and strong antimicrobial activity and effective tissue adhesion provides a significant advantage over conventional administration routes [227]. Li and colleagues developed a smart core–shell PCMAT hydrogel with adenine-loaded MIL-88b-NH_2_ nanocages, providing stage-responsive wound management and >80% antimicrobial activity within 6 h. The optimum PCMAT-0.3 had a reduction in pore size to ~13.5 µm under endogenous conditions, and the in vivo evaluation showed significantly improved wound healing by day 14, highlighting the potential of this material for responsive wound therapy [228]. In another study, Ashfaq and colleagues developed a pH- and ion-responsive hydrogel containing Bergenia ciliata extract and silver nanoparticles, showing strong swelling responsiveness to different wound-relevant conditions. In vivo, the BCNP-loaded hydrogel decreased diabetic wound contraction and showed pro-antioxidant and anti-inflammatory effects, re-epithelialization, neovascularization, and collagen deposition, thereby demonstrating its potential for diabetic wound healing [229]. Tan et al. developed a protease-responsive hydrogel comprising oxidized hyaluronic acid, EGCG/GelMA, and resveratrol nanoparticles to deliver polyphenols on demand in diabetic wounds. Protease-triggered release decreased oxidative stress and inflammation, increased polarization of macrophages to a healing phenotype and collagen deposition, and supported diabetic wound healing in vivo [230]. Table 2 presents a comparative summary of major nanomaterial classes for wound healing, highlighting biological activities, key signaling pathways, antimicrobial and antioxidant properties, angiogenic potential, and current stages of development.

## 6. Bottlenecks and Safety Considerations Regarding Nanomaterials in Targeted Wound Healing

Despite their therapeutic advantages, nanomaterial-based wound-healing systems also present important platform-specific limitations. Metallic and metal oxide nanoparticles can have potent antimicrobial, antioxidant, photothermal, or pro-regenerative properties, while polymeric, lipid-based, and hydrogel systems can typically be used as matrices for controlled delivery of therapeutic agents, can be biodegradable, conform to tissue surfaces, and can carry biological therapeutics. However, these benefits are countered by concerns about particle aggregation, particle size and surface charge variability, low drug-loading capacity, premature drug release, low mechanical stability, or low retention at the wound site. Significantly, the biological response to nanomaterials is highly dependent on particle size and morphology, surface chemistry and composition, dose, degradation rate, and interactions with the wound environment [41]. At high concentrations, metal and metal-oxide nanomaterials can induce high levels of ion dissolution and ROS production, leading to oxidative stress, mitochondrial dysfunction, membrane damage, and decreased cell viability. This does not necessarily imply increased cellular uptake and reactivity of smaller nanoparticles, but it is important to note that both uptake and reactivity are strongly dependent on surface characteristics and material composition. Furthermore, incomplete degradation can lead to persistence in tissues, and released nanoparticles or degradation products can be distributed systemically and interact with immune cells, leading to inflammatory or immunogenic responses. Therefore, short-term cell-viability assays should not be considered sufficient evidence of long-term biosafety [43].

Another disadvantage is the ability to reproduce and compare the results reported. Due to the variety of different procedures used for the synthesis of nanoparticles, different sizes of nanoparticles, different surface modifications, different loading levels of drug, different doses, wound models, bacterial strains, different irradiation conditions, and different evaluation periods, it is difficult to make direct comparisons between different formulations. Synthesis or processing conditions can differ slightly, which may influence particle size distribution, surface chemistry, aggregation state, drug release, ion dissolution, and subsequently, the biological activity [46]. Likewise, positive outcomes seen in simplified in vitro models and short-term animal models may fail to adequately reflect the complexity of the clinical wound environment, characterized by the presence of variations in wound depth, wound exudates, bacterial load, tissue perfusion, inflammation, and patient-specific comorbidities. Standardization of critical quality attributes such as particle size and size distribution, surface charge, morphology, composition, drug loading/encapsulation efficiency, drug release, degradation behavior, and dose-normalized biological activity would therefore benefit reproducibility. Additional studies of longer duration, including dose–response, pharmacokinetic, and biodistribution studies, clearance, degradation, histopathology, immune response, and tissue-remodeling endpoints, are necessary to determine whether short-term regenerative effects result in sustained therapeutic benefit. Additionally, the intensity, duration, temperature distribution, and frequency of stimulation should be standardized in systems that are responsive to light and heat, or to other stimuli, because increasing or prolonging the treatment may cause damage to other tissue [231].

Another big hurdle is manufacturing and regulatory translation. Scale-up, sterilization, storage solutions, and batch-to-batch production may prove to be challenging when the chemical and physical properties of the nanomaterials are not well controlled in the lab. Different sizes, aggregation, and functionalization of the particles, drug loading, or release profiles can directly impact efficacy and safety. Sterilization may also result in changes to the structure of the polymer, aggregation of the nanoparticles, surface coating, or release characteristics of the encapsulated therapeutics; long-term storage is likely to cause changes to the surface coating or to the release characteristics of the encapsulated therapeutics. Regulatory evaluation of nanotechnology-based wound therapeutics can be complex, as the biological activity can be provided by the nanocarrier or the therapeutic cargo or a combination of the two, especially when the therapeutic cargo is multifunctional [232]. It will therefore be critical to develop consistent critical quality attributes (CQAs), validated manufacturing processes, clinically relevant and scientifically appropriate stability specifications, and safety endpoints. Persistent nanomaterials should also be considered with regard to environmental issues, especially their environmental release from manufacturing, clinical use, and disposal. Therefore, clinical translation needs to be based on a formulation-specific risk–benefit analysis that takes into account therapeutic efficacy, physicochemical stability, toxicological profile, biodistribution, biodegradation, and manufacturing reproducibility, as well as regulatory requirements, instead of just short-term biocompatibility or wound-closure results.

## 7. Clinical Translation and Current Human Evidence

While most of the research on wound healing using nanomaterials is still preclinical, limited clinical studies have started to investigate the use of wound dressings using nanomaterials in humans. For instance, NCT07147790 was conducted to study the use of a spray containing silver nanoparticles combined with negative-pressure wound therapy in post-revascularization diabetic foot wounds, and clinical outcomes included complete epithelialization, reduction in wound size, infection rate, inflammatory markers, pain, and longer-term follow-up. Likewise, NCT04834245 tested a hydrogel/nano-silver dressing in patients with DFUs, and NCT02108535 tested nanocrystalline silver against 1% silver sulfadiazine in 100 adults with second-degree burns, in a Phase 4 randomized trial. A more recent study (NCT07521176) investigated a nanocrystalline silver membrane for postoperative wound healing and re-epithelisation, but this study was conducted with a small sample size (*n* = 12) and in a specific periodontal setting, which limits the findings to chronic or complex skin wounds. These studies highlight that the translation of nanotechnology-enabled wound dressings into human studies is taking place but is very limited compared with the amount of in vitro and animal research available. Table 3 provides clinical translation of nanomaterial-based wound-healing systems, including registered clinical trials, wound indications, intervention, study status, and translational relevance.

However, other challenges exist that hinder clinical translation, including maintaining the physicochemical characteristics of the nanomaterial throughout scale-up, sterilization, packaging, and storage. Size, aggregation, surface chemistry, silver-ion release, drug loading, and degradation are some of the variables that can vary between different batches and can affect efficacy and toxicity. Furthermore, long-term clinical assessment should include, among other things, systemic exposure, accumulation in tissue, repeated-use toxicity, local inflammatory response, and the possible effects of persistent nanomaterials. There is a potential need for a regulatory assessment of the nanotechnology and the dressing system as a whole, from a regulatory perspective, for wound products that contain nanomaterials and are intended for use against microbes (antimicrobial activity) or to deliver therapeutic agents (therapeutic drug delivery). However, it is also crucial to have post-market evidence, such as NCT05045430, which is a multicentre post-market surveillance study to evaluate the safety and performance of a silver-containing non-woven dressing in chronic and acute wounds. Despite the fact that a number of wound products containing silver and/or nano-silver have reached the clinical testing phase, clinical translation of multifunctional nanocarriers, stimuli-responsive systems, nanozymes, graphene-based platforms, and targeted nanotherapeutics has been limited, with a significant amount of work remaining to ensure long-term safety, scalable manufacturing, regulatory consistency, and cost-effective commercialization.

## 8. Challenges and Future Perspectives

Although the engineering of multifunctional nanomaterials for skin regeneration has made significant progress, several critical challenges still limit their clinical translation [233]. These challenges become particularly important in pathway-directed wound nanomedicine, because signaling pathways do not have a constant biological function throughout the healing process. Wound healing is a dynamic and overlapping process involving inflammation, proliferation, angiogenesis, re-epithelialization, and tissue remodeling, and the intensity and function of individual signaling pathways change according to the healing stage and cellular context [234,235]. For example, early activation of inflammatory pathways such as NF-κB and MAPK is essential for immune-cell recruitment, antimicrobial defence, cytokine production, and initiation of tissue repair; however, their persistent activation can maintain chronic inflammation, oxidative stress, and tissue damage. Similarly, TGF-β/Smad signaling is indispensable for fibroblast activation, extracellular matrix deposition, wound contraction, and normal tissue repair, but excessive or prolonged TGF-β activity can promote myofibroblast differentiation, excessive collagen deposition, and pathological fibrosis [236,237]. Therefore, continuous inhibition of inflammatory or TGF-β signaling may also impair normal healing, whereas uncontrolled activation may favour chronic inflammation or scar formation. This highlights a major limitation of conventional nanocarriers that provide continuous drug exposure without considering the changing biological requirements of different healing phases. Future pathway-directed nanomedicines should consequently focus on dose-, time-, and cell-specific modulation, in which the therapeutic intensity is adjusted according to the wound stage and the targeted cellular population. This is particularly important because the same pathway may produce different or even opposing effects in macrophages, fibroblasts, keratinocytes, endothelial cells, and stem/progenitor cells. Thus, ligand-mediated targeting, biomimetic surfaces, cell-responsive carriers, and selective intracellular delivery may be required to regulate the desired cell population while minimizing off-target pathway modulation [238,239].

A second major challenge relates to achieving precise spatiotemporal regulation of pathway activity within the heterogeneous wound microenvironment. Chronic and diabetic wounds contain spatially distinct regions characterized by persistent inflammation, hypoxia, excessive reactive oxygen species, infection, abnormal pH, protease overexpression, impaired vascularization, and defective extracellular matrix remodeling [239,240]. Consequently, a uniform and sustained release profile may expose healthy or already-resolving regions to unnecessary drug concentrations while failing to provide sufficient therapy at highly pathological sites. Stimuli-responsive nanomaterials that respond to endogenous signals such as pH, ROS, glucose, hypoxia, or enzymes, or to external triggers such as light, ultrasound, magnetic fields, temperature, or electrical stimulation, may provide more appropriate on-demand and localized release [240,241]. Importantly, future systems should progress beyond single-trigger release toward sequential or multi-stimuli-responsive platforms capable of adapting to the changing wound environment. For example, a nanocarrier could initially support controlled inflammatory signaling or antimicrobial activity, subsequently suppress excessive inflammation, and later reduce pathological TGF-β-driven fibroblast activation during remodeling. Such an approach would more closely reproduce the physiological sequence of wound healing than continuous activation or inhibition of a single pathway. Furthermore, wound signaling is controlled by interconnected networks rather than isolated pathways; TGF-β/Smad, PI3K/Akt, Wnt/β-catenin, NF-κB, MAPK, JAK/STAT, Nrf2, and angiogenic signaling can interact through feedback and compensatory mechanisms [242,243]. Therefore, targeting one pathway may unintentionally influence other pathways and produce unpredictable outcomes. Consequently, future studies should evaluate pathway crosstalk, downstream functional responses, dose–response relationships, and cellular specificity rather than relying only on changes in individual protein or gene markers. The development of such systems is further complicated by long-term nanomaterial safety, cytotoxicity, tissue accumulation, biodegradation, batch-to-batch variability, and scalability, because small changes in particle size, morphology, surface chemistry, functionalization, or drug loading can markedly alter biological behavior [244].

From a translational and future perspective, there is therefore a need to move from static nanomaterial-based delivery toward intelligent, adaptive, and phase-specific wound nanomedicine. Clinically relevant human skin models, 3D bioprinted tissues, organ-on-chip platforms, advanced ex vivo models, and appropriate large-animal models should complement conventional small-animal studies to better reproduce human skin architecture, immune responses, wound contraction, and scar formation. Standardized protocols for nanomaterial characterization, toxicity, biodistribution, biodegradation, sterilization, manufacturing, storage stability, and regulatory evaluation will also be essential for reproducible clinical development [244]. In parallel, artificial intelligence (AI) and machine learning (ML) offer considerable potential for addressing the complex multidimensional relationships between nanomaterial properties, wound microenvironment, signaling pathways, and therapeutic outcomes [39,245]. AI/ML models can integrate physicochemical, transcriptomic, proteomic, metabolomic, and single-cell datasets to identify pathway crosstalk, predict structure–property–function relationships, optimize drug loading and release kinetics, and determine the most appropriate combination of dose, timing, cellular target, and therapeutic pathway [245]. Such approaches may also support the development of personalized wound treatments by predicting which signaling networks are dysregulated in individual patients and selecting nanocarriers capable of responding to patient-specific pathological cues. Ultimately, integration of pathway-directed nanocarriers with AI-guided design, stimuli-responsive release, 3D bioprinting, stem-cell therapy, and extracellular-vesicle-based approaches may enable therapeutic systems that dynamically respond to the evolving wound environment. The central objective should therefore not be to activate or inhibit a particular signaling pathway, but to achieve the right pathway activity, at the right dose, in the right cell, at the right location, and at the right stage of healing, thereby promoting regeneration while minimizing fibrosis and other unintended effects.

## 9. Conclusions

Advanced multifunctional nanomaterials have emerged as platforms that simultaneously deliver therapeutics and modulate key signaling pathways that regulate wound healing, providing a rational approach to scarless skin regeneration. Modulating interconnected pathways such as TGF-β/Smad, Wnt/β-catenin, NF-κB, PI3K/Akt, and MAPK can not only inhibit chronic inflammation but also stimulate angiogenesis, inhibit fibroblast-to-myofibroblast transition, and coordinate extracellular matrix remodeling, thereby shifting fibrotic repair towards regenerative healing. The inclusion of stimuli-responsive and biomimetic elements allows these systems to dynamically respond to the evolving wound microenvironment for spatiotemporal control of drug release and cellular responses, further optimizing therapeutic precision and efficacy. The versatility of these nanotechnologies is further enhanced by the development of hybrid nanoplatforms, such as nanogels, lipid-based carriers, and metal–organic frameworks, which integrate multiple functions to coordinate with complex wound pathophysiology; these innovations facilitate not only accelerated wound closure but also the restoration of skin architecture with minimal scarring due to reduced collagen disorganization and limited excessive myofibroblast activity. However, there are still challenges in terms of scalability, long-term safety, and clinical translation, and interdisciplinary efforts will be needed to overcome these hurdles; therefore, continued integration of nanotechnology with systems biology and emerging approaches such as AI-driven design and advanced biofabrication will further refine and improve these strategies and ultimately result in the development of next-generation personalized wound-healing therapies.

## Figures and Tables

**Table 2:** Comparative summary of major nanomaterial classes for wound healing.

Nanomaterial Class	Representative Examples	Biological Activity (Overall)	Key Signaling Pathways Affected	Antimicrobial Properties	Antioxidant Activity	Angiogenic Potential	Stage of Development
Metal and metal oxide nanoparticles	AgNPs, AuNPs, ZnO-NPs, Cu/CuO-NPs, CeO_2_-NPs, Se-NPs	Broad-spectrum antimicrobial, anti-inflammatory, pro-proliferative, pro-migratory, and immunomodulatory	NF-κB ↓; PI3K/Akt ↑; MAPK/ERK ↑; TGF-β/Smad ↑; HIF-1α/VEGF ↑; Nrf2 ↑	Strong; membrane disruption, ROS generation, and metal-ion release	Moderate–strong; ROS scavenging or controlled ROS generation	Moderate–strong; enhanced VEGF expression and neovascularization	Extensive in vitro/in vivo studies; limited early-phase clinical studies, particularly Ag-based dressings
Carbon-based nanomaterials	Graphene, GO, rGO, CNTs, carbon dots, nanodiamonds	Antimicrobial, antioxidant, pro-adhesive, pro-migratory and immunomodulatory	VEGF ↑; TGF-β/Smad ↑; PI3K/Akt ↑; MAPK ↑; NF-κB modulation	Moderate–strong; membrane disruption and biofilm inhibition	Strong; ROS scavenging and radical quenching	Moderate; increased endothelial proliferation, VEGF expression, and neovascularization	Predominantly in vitro/in vivo; limited clinical translation
Polymeric nanomaterials	Chitosan NPs, PLGA NPs, silk fibroin NPs, PEG/PEG-PLA NPs, dendrimers	Biocompatible, anti-inflammatory, pro-proliferative and suitable for controlled drug/growth-factor delivery	PI3K/Akt ↑; MAPK/ERK/JNK/p38 ↑; TGF-β/Smad ↑; Wnt/β-catenin ↑; NF-κB ↓; VEGF/HIF-1α ↑	Weak–moderate intrinsically; chitosan shows greater activity; often enhanced by antimicrobial cargo	Weak–moderate; mainly dependent on loaded antioxidants such as curcumin or quercetin	Moderate–strong when loaded with VEGF, Cu ions, or other pro-angiogenic factors	Extensive in vitro/in vivo research; selected chitosan- and PLGA-based systems in early clinical/dermal evaluation
Lipid-based nanocarriers	Liposomes, solid lipid NPs, nanostructured lipid carriers, exosomes	Biocompatible, anti-inflammatory, pro-regenerative, and efficient cargo delivery	PI3K/Akt ↑; MAPK ↑; TGF-β/Smad ↑; VEGF/HIF-1α ↑; NF-κB ↓; exosomes additionally modulate miRNA-related pathways	Weak intrinsically; mainly achieved through loaded antibiotics, peptides, or essential oils	Moderate when loaded with vitamins, polyphenols, or antioxidant enzymes	Moderate; exosomes and VEGF-loaded liposomes can enhance angiogenic responses	Mainly in vitro/in vivo; exosome approaches remain largely preclinical; limited early clinical evaluation
Nanofibers and nanocomposite hydrogels	Electrospun PCL, PLGA, gelatin, chitosan and PVA nanofibers; NP-loaded nanocomposite hydrogels	ECM-mimetic, hemostatic, antimicrobial when loaded, pro-migratory and pro-proliferative	TGF-β/Smad ↑; PI3K/Akt ↑; MAPK ↑; VEGF/HIF-1α ↑; NF-κB modulation	Moderate–strong when incorporated with Ag, Cu, ZnO, antibiotics, or essential oils	Moderate when incorporating CeO_2_, Se, or antioxidant phytochemicals	Moderate–strong; porous architecture supports endothelial infiltration and neovascularization	Extensive in vitro/in vivo evidence; several systems have reached clinical/commercial advanced-dressing applications
Bioinspired and stimuli-responsive nanocomposites	Enzyme-responsive hydrogels, ROS/pH/temperature-responsive nanogels, cell-mimetic nanocarriers, MOF-based systems	Multifunctional antimicrobial, antioxidant, anti-inflammatory, pro-angiogenic and capable of on-demand drug release	NF-κB, PI3K/Akt, MAPK, TGF-β/Smad, Wnt/β-catenin, HIF-1α/VEGF and Nrf2, depending on formulation	Tunable; moderate–strong when combined with metal NPs, MOFs or antimicrobial peptides	Tunable; moderate–strong, particularly in nanozyme- or antioxidant-loaded systems	Moderate–strong through controlled release of pro-angiogenic factors	Predominantly in vitro/in vivo; largely preclinical with very limited clinical translation

Note: ↑ indicates pathway activation or upregulation, whereas ↓ indicates pathway inhibition or downregulation.

**Table 3:** Clinical trials and translational evidence of nanomaterial-based wound-healing systems.

Clinical Trial	Nanomaterial/Intervention	Wound Indication	Status Design	Key Relevance	Reference
NCT07147790	Silver nanoparticle spray + NPWT	Post-revascularization diabetic foot wounds	Clinical comparative study; 88 patients included in final analysis	Directly relevant to silver nanoparticle-based wound therapy; outcomes included complete epithelialization, wound-size reduction, infection rate, CRP, and pain, with follow-up extending to 6 months	https://clinicaltrials.gov/study/NCT07147790, accessed on 6 July 2026
NCT04834245	Hydrogel/nano-silver dressing	Diabetic foot ulcers	Randomized prospective study; 30 patients; completed	Compared hydrogel/nano-silver dressing with conventional dressing and evaluated ulcer-size reduction over 3 weeks.	https://clinicaltrials.gov/study/NCT04834245, accessed on 6 July 2026
NCT07521176	Nano-crystalline silver membrane	Postoperative gingival wound healing	Randomized split-mouth study; 12 participants; completed	Evaluated tissue healing and re-epithelialization weekly for 4 weeks after gingival depigmentation.	https://clinicaltrials.gov/study/NCT07521176, accessed on 6 July 2026
NCT02108535	Nanocrystalline silver dressing (Acticoat)	Second-degree burns	Phase 4 randomized controlled trial; 100 participants; completed	Compared nanocrystalline silver with 1% silver sulfadiazine; assessed epithelialization, pain, infection, adverse reactions, and treatment cost.	https://clinicaltrials.gov/study/NCT02108535, accessed on 6 July 2026
NCT01598480	Nano-silver impregnated activated-carbon dressing	Superficial dermal burns	Clinical trial; completed	Evaluated wound-healing effects of a silver-particle-containing activated-carbon dressing in burn patients.	https://clinicaltrials.gov/study/NCT01598480, accessed on 6 July 2026
NCT05045430	Silver II non-woven dressing	Chronic and acute wounds, including diabetic ulcers, burns and surgical wounds	Post-market surveillance; multicenter; estimated enrollment 240	Useful for discussing clinical safety, performance, and commercialization/post-market evidence, although this should not be described as a nanoparticle trial unless the product composition specifically supports that claim.	https://clinicaltrials.gov/study/NCT05045430, accessed on 6 July 2026

## Data Availability

No new data were created or analyzed in this study.

## Abbreviations

The following abbreviations are used in this manuscript:

ECM	Extracellular matrix
ROS	Reactive oxygen species
TGF-β	Transforming growth factor-Beta
NF-κB	Nuclear factor Kappa-B
PI3K	Phosphoinositide 3-kinase
Akt	Protein kinase B
MAPK	Mitogen-activated protein kinase
HIF-1α	Hypoxia-inducible factor-1 Alpha
VEGF	Vascular endothelial growth factor
YAP	Yes-associated protein
AgNPs	Silver nanoparticles
AuNPs	Gold nanoparticles
ZnO NPs	Zinc oxide nanoparticles
CuNPs	Copper nanoparticles
PVA	Poly(vinyl alcohol)
PEG	Poly(ethylene glycol)
SEM	Scanning electron microscopy
TEM	Transmission electron microscopy
FTIR	Fourier transform infrared spectroscopy
XRD	X-ray diffraction
